# Aregs-IGFBP3-mediated SMC-like cells apoptosis impairs beige adipocytes formation in aged mice

**DOI:** 10.1016/j.molmet.2025.102125

**Published:** 2025-03-19

**Authors:** Shifeng Wang, Yuanxu Cui, Limei Wang, Chun Feng, Yifei Sun, Bangyun Huo, Honglu Jiang, Mingyu Zhao, Yingying Tu, Qiyue Wang, Yutao Yang, Qiang Zhang

**Affiliations:** 1 Animal Zoology Department, Kunming Medical University, Kunming, 650000, China; 2 Department of Emergency, The First Affiliated Hospital of Kunming Medical University, Kunming, 650032, China; 3 Science and Technology Achievement Transformation Center, Kunming Medical University, Kunming, 635000, China; 4 School of Anesthesiology, Zunyi Medical University, Zunyi, 563000, China; 5 Department of Otolaryngology, The First People's Hospital of Yunnan Province, Kunming, 650000, China; 6 Department of Urology, the Second Affiliated Hospital of Kunming Medical University, Kunming, 650000, China; 7 The First School of Clinical Medicine, Kunming Medical University, Kunming, 650000, China

**Keywords:** Aregs, IGFBP3, SMC-Like cells, Browning, Aging

## Abstract

Aging is associated with a decline in the browning capacity of white adipose tissue (WAT), contributing to metabolic dysfunction. Beige adipocytes, which dissipate excess energy as heat, are a key feature of this process. In this study, we investigate the role of adipose stem and progenitor cells (ASPCs), specifically the Aregs (CD142+) subpopulation, in regulating beige adipocyte formation in aged mice under cold stimulation. Our findings reveal that Aregs significantly increase in the subcutaneous WAT (sWAT) of aged mice following cold exposure. We further demonstrate that Aregs secrete insulin-like growth factor binding protein 3 (IGFBP3), which appears to play a pivotal role in the cross-talk between adipogenesis-regulatory cells (Aregs) and smooth muscle cell-like (SMC-like) cells, thereby leading to the inhibition of beige adipocytes formation. Functional enrichment analysis highlighted the activation of TGFβ, MAPK and p53 signaling pathways in SMC-like cells, all of which are known to induce cell apoptosis and fibrosis. Moreover, IGFBP3 was found to interact with receptors and signaling molecules, including Egfr, Irf1 and Cdkn1a, in SMC-like cells, enhancing their apoptosis. Co-culture experiments confirmed that IGFBP3 significantly suppressed the formation of beige adipocytes, further corroborating its role in impairing browning. Overall, our study provides novel insights into the molecular mechanisms by which Aregs and IGFBP3 contribute to the age-related decline in WAT browning. These findings suggest potential therapeutic targets for reversing impaired WAT browning in aging and related metabolic disorders.

## Highlights

• Aregs accumulation in aged mice under cold exposure.
• IGFBP3 secreted by Aregs induces apoptosis in SMC-like cells.
• Aregs-IGFBP3 signaling pathway impairs beige adipocyte formation.

## Graphical abstract

**Figure:**
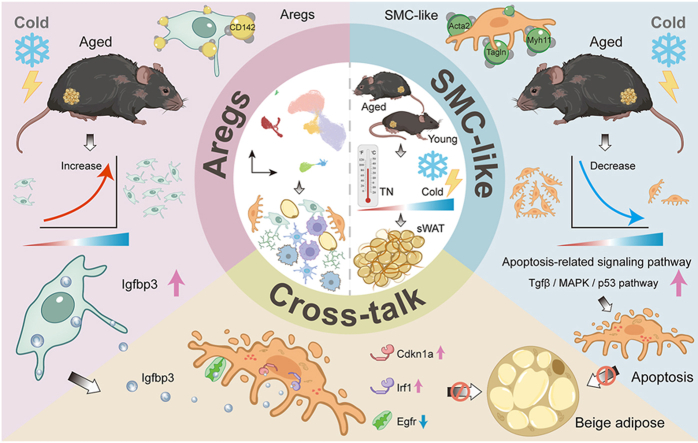

## 1 Introduction

Adipose tissue plays a pivotal role not only in energy storage but also as an active endocrine organ regulating systemic metabolism. Among its subtypes, brown adipose tissue (BAT) and beige adipose tissue have garnered attention for their unique ability to dissipate energy via thermogenesis, mediated by mitochondrial uncoupling through uncoupling protein 1 (UCP1) [1,2]. Beige adipose tissue, in particular, has drawn significant interest due to its remarkable plasticity. It can transdifferentiate from widespread white adipose tissue (WAT) or de novo differentiate from beige progenitor cells scattered within WAT under specific stimuli, such as classical cold exposure. De novo differentiated beige progenitor cells exhibit smooth muscle-like (SMC-like) characteristics [[3], [4], [5]]. This entire process, referred to as “browning” [6,7], holds great potential for combating obesity, cardiovascular diseases (CVD), and other metabolic disorders [[8], [9], [10]].

However, the ability of adipose tissue to undergo browning declines with aging. Aged individuals exhibit a reduced capacity for beige fat formation, which is often associated with impaired thermogenesis and metabolic dysfunction [11,12]. This decline is thought to contribute to the increased risk of obesity, insulin resistance, and CVD observed in the elderly [12,13]. The diminished browning capacity in aging fat depots has been linked to changes in the adipose tissue microenvironment, including altered stem cell function [14,15], the accumulation of inflammatory mediators [8] and a reduced response to cold exposure [16]. Understanding the cellular and molecular mechanisms behind this decline in browning ability could provide valuable insights into how aging exacerbates metabolic diseases and identify potential therapeutic targets to reverse these age-related changes.

Adipogenesis-regulatory cells (Aregs), a CD142+ subpopulation of adipose stem and precursor cells (ASPCs), were first identified and molecularly characterized in 2018 by Schwalie et al. These cells are refractory to adipogenesis and exhibit a paracrine capacity to suppress adipocytes formation in vitro and in vivo, suggesting a critical role in regulating adipose tissue plasticity and metabolic functions [17]. A recent study has suggested that Aregs may play a pivotal role in the remodeling of WAT under cold stimulation [18]. Additionally, it has been shown that the anti-adipogenic activity of Aregs increases with aging, which may contribute to the age-related decline in adipose tissue function and thermogenic capacity [19].

Insulin-like growth factor binding protein 3 (IGFBP3) has been identified as a critical mediator secreted by Aregs in this study. It is well known for regulating cell proliferation and differentiation by modulating the bioavailability of insulin-like growth factors (IGFs). Specifically, IGFBP3 binds to IGFs, inhibiting its interaction with the IGFs receptors, thereby reducing IGFs signaling activity [[20], [21], [22], [23]]. In fact, IGFBP3 is also a known inhibitor of adipogenesis and can induce adipocytes apoptosis [24].

These studies underscore the remarkable plasticity of Aregs in modulating the adipose tissue microenvironment across different ages and conditions. However, the roles of Aregs and IGFBP3 in the impaired browning capacity of aging adipose tissue remains unexplored. Understanding whether Aregs and IGFBP3 are involved in the diminished browning capacity of aged adipose tissue under cold exposure, and the underlying molecular mechanisms, is crucial for future research and warrants further investigation. In this study, we further analyze Aregs cells and beige adipocytes precursor SMC-like cells using publicly available single-cell RNA sequencing (scRNA-seq) data (GEO: GSE227439). Through integrated analysis and experimental validation, we present the first detailed description of the increased presence of the Aregs subpopulation in aged mice under cold stimulation. We demonstrate that Aregs inhibit the number of SMC-like cells through the secretion and action of IGFBP3, leading to a novel mechanism of impaired browning ability in response to cold stimulation in aged mice.

## 2 Materials and methods

### 2.1 Animal experiments

All animal procedures received approval from the Animal Care and Use Committee of Kunming Medical University (Approval No. KMMU-20230939) and conducted in compliance with institutional guidelines. Male C57BL/6 mice, classified as young (4 weeks) and old (52 weeks), were obtained from the Experimental Animal Center of Kunming Medical University. Mice were housed under controlled conditions at 30 °C for 3 weeks to ensure acclimatization. Following this period, they were either maintained at thermoneutrality (30 °C) for an additional 14 days (TN group) or subjected to cold exposure at 6 °C for 14 days (cold group). Throughout the experiment, all mice were provided ad libitum access to standard chow and maintained under a 12 h-light/dark cycle.

### 2.2 Primary adipose stem and progenitor cells (ASPCs) isolation

Murine sWAT was dissected from 9-week-old male and female mice separately into ice-cold PBS with 1% (v/v) penicillin-streptomycin solution (P/S) (Gibco, 15070063). The tissue was minced with scissors and digested in DMEM (Gibco, 11965092) containing 1 mg/mL collagenase type I at 37 °C with constant shaking at 1200 rpm for 90 min. After digestion, the tissue was filtered through a 70 μm nylon mesh (Absin, abs7232) and centrifuged at 1200 rpm for 10 min. The floating lipid layer was carefully removed, and the stromal vascular fraction (SVF) pellet was resuspended in red blood cell lysis buffer (Gibco, 00-4333-57). The recovered cells were washed with pre-warmed PBS (Gibco, 10010023) containing 3% fetal bovine serum (FBS) (Gibco, 10270-106). Finally, primary ASPCs were cultured in complete medium (DMEM supplemented with 10% FBS and 1% P/S) in a CO_2_ incubator.

### 2.3 Lentiviral transduction

We used a lentivirus-based CRISPR/Cas9 system to knockout (KO) Igfbp3 in Aregs. The lentivirus employed two gRNAs (gRNA1: CTGACTTGCGCGCTGCGCGA, gRNA2: CCGCGGAGCAGTACCCGCTG) targeting the full-length sequence of Igfbp3. Additionally, we included a control group of Aregs treated with non-targeting CRISPR (KO ctrl) using a scramble gRNA (GGTGTAGTTCGACCATTCGTGGTTTTAGAGCTAGAAATAGCAAGTTAAAATAAGGCTAGTCCGTTATCAACTTGAAAAAGTGGCACCGAGTCGGTGC) (Ubigene, Guangzhou). Briefly, the lentivirus, along with the lentiviral transduction enhancer Lentiblast reagent (OZ Biosciences, LBPX500), was used to infect ASPCs. 2 days post-infection, transduced cells were selected with puromycin (1 μg/mL). Surviving cells were then used for subsequent experiments.

### 2.4 Browning differentiation

Fully confluent ASPCs were induced to differentiate by switching to differentiation medium I, which consisted of DMEM complete medium supplemented with 850 nM insulin (Solarbio, I8830), 0.5 mM isobutylmethylxanthine (Solarbio, II0010), 1 μM dexamethasone (Solarbio, ID0170), 125 nM indomethacin (Solarbio, SI9020), 1 μM rosiglitazone (Solarbio, IR0130), and 1 nM 3,3′,5-triiodo-l-thyronine (T3) (MCE, HY-A0070A) for 2 days. Following this, the cells were transferred to differentiation medium II, which contained DMEM complete medium supplemented with 850 nM insulin, 1 μM rosiglitazone, and 1 nM T3, and incubated for an additional 6 days.

### 2.5 RNA isolation and quantitative PCR (qPCR)

Cells were harvested using pre-chilled TRIzol reagent (Invitrogen, 15596018), and total RNA was extracted following the chloroform-isopropanol-ethanol method according to the manufacturer's protocol. For sWAT, samples were first minced using a TissueLyser II (Qiagen, 85300), and RNA extraction was performed similarly to the cellular RNA extraction procedure. One microgram of RNA was reverse transcribed into complementary DNA (cDNA) using the MightyScript Plus First Strand cDNA Synthesis Master Mix (Sangon Biotech, B639252). qPCR was conducted using SGExcel FastSYBR Mixture (Sangon Biotech, B532955) and the ABI PRISM™ 7900HT system (Applied Biosystems). Primer sequences are provided in (Supplementary Table 1). Relative mRNA expression levels were calculated accordingly using the 2^−ΔCt^ method and normalized to GAPDH.

### 2.6 Immunohistochemistry fluorescence

sWAT was carefully excised and fixed in 4% paraformaldehyde (Beyotime, P0099) for 18–24 h. The tissue was then immersed in phosphate-buffered sucrose solutions (10%, 20%, and 30%) until it sank. After removing excess moisture with absorbent paper, the samples were embedded in O.C.T. compound (Sakura Finetek, TISSUE-TEK 4583), fully covered with additional O.C.T., and flash-frozen in isopentane and liquid nitrogen for ∼30 s. The frozen samples were equilibrated for 5 min in a cryostat chamber before sectioning into 15 μm slices using a Leica CM1850 cryostat. The sections were mounted on adhesive glass slides, air-dried, stored at −20 °C, and stained and analyzed promptly. For staining, sections were thawed, washed with PBST, and blocked for 2 h at room temperature (RT) with 5% bovine serum albumin (BSA) (sigma Aldrich, A7030) and 0.1% Triton X-100 in PBS. Primary antibodies (UCP1, 1:100, CST, 72298; CD142, 1:100, R&D Systems, AF3178) were applied and incubated overnight at 4 °C. After washing with PBST, sections were incubated for 3 h at RT in the dark with anti-goat FITC secondary antibody (1:400, Proteintech, SA00003-3) for CD142, anti-rabbit CoraLite594 secondary antibody (1:400, Proteintech, SA00013-8) for UCP1, and DAPI (Invitrogen, D1306). Immunofluorescence images were captured using a Nikon TiE-A1 Plus confocal microscope (Nikon, Japan).

### 2.7 Immunofluorescence staining

Primary adipocytes were stained with MitoTracker™ Deep Red FM (Invitrogen, A66440) to label mitochondria, diluted in pre-warmed serum-free DMEM, and incubated for 30 min at 37 °C. And then the adipocytes were fixed in 4% paraformaldehyde at RT for 15 min, followed by 0.3% Triton™ X-100 (Sigma, T8787) for 10 min, and then blocked in 1% BSA for 1 h at RT. Next, adipocytes were subjected to an overnight incubation at 4 °C with CD142 primary antibody (1:100, R&D Systems, AF3178). Subsequently, adipocytes were stained with anti-goat FITC secondary antibody (1:400, Proteintech, SA00003-3) for 1 h at RT. Then, the coverslips were mounted with mounting reagent including DAPI. Finally, the coverslips were imaged using confocal microscope (Nikon, Japan).

### 2.8 Immunoblotting

Primary adipocytes were scraped, and adipose tissue was homogenized in cold RIPA buffer supplemented with a protease inhibitor cocktail (Roche, 11836170001) on ice for 1 h. The lysates were centrifuged at 12,000 rpm for 15 min at 4 °C, and the supernatant protein concentration was measured using the BCA Protein Assay (Thermo Fisher Scientific, 23225). Protein samples (20 μg) were mixed with 4 × loading buffer, heated at 95 °C for 5 min, and separated on a 15% SDS-PAGE gel. Proteins were transferred to 0.45 μm nitrocellulose (NC) membranes (GE Healthcare, 10600002), blocked with 5% non-fat milk in TBS for 1 h, and incubated overnight at 4 °C with primary antibodies: anti-CD142 (1:1000, R&D Systems, AF3178), anti-IGFBP3 (1:1000, Proteintech, 10189-2-AP), anti-UCP1 (1:1000, CST, 72298S), anti-TOMM20 (1:1000, CST, D8T4N), anti-P-SMAD2/3 (1:1000, CST, 8828S), anti-SMAD2/3 (1:1000, CST, 8685S), anti-P-p38 (1:1000, CST, 4511S), anti-p38 (1:1000, CST, 8690S), anti-p53 (1:1000, CST, 30313S), anti-p38 (1:1000, Proteintech, 28248-1-AP) and anti-GAPDH (1:1000, CST, 97166). After washing with TBST, membranes were incubated with secondary antibodies for 1 h at RT. Protein bands were detected using Pierce ECL substrate (Thermo Fisher Scientific, 32106) and visualized with a BioLight Gelview 6000 Pro imaging system.

### 2.9 Flow cytometry

To isolate the CD142+ cell population from mouse ASPCs, flow cytometry was performed. Briefly, ASPCs were collected and resuspended in FACS buffer (Biolegend 420201). After filtering through a 70 μm nylon mesh (Absin, abs7232), the cell density was adjusted to 1 × 10^ˆ6^ cells/mL. Cells were incubated with anti-CD142 antibody (1:50, R&D Systems, AF3178) at 4 °C for 1 h. Following washing with FACS buffer, cells were incubated with anti-goat FITC secondary antibody (1:200, Proteintech, SA00003-3) at 4 °C for 30 min, followed by additional washing with FACS buffer. Cell sorting was conducted using a BD FACSAria™ flow cytometer (BD Biosciences), equipped with a 100 μm nozzle and lasers/filters for FITC detection (488 nm and 515/20 nm).

### 2.10 scRNA-seq data preprocessing and quality control

Raw data for GSE227439 were downloaded from the GEO database and processed using CellRanger (version 7.0.1) from 10× Genomics. Sequence alignment was performed against the mm10 genome using the CellRanger count module with default parameters. To ensure data quality, low-quality cells were excluded based on the following criteria: (1) total UMI count <200; (2) total UMI count >5000; and (3) mitochondrial transcript proportion >15%.

### 2.11 Transwell co-culture of Aregs and non-Aregs cells

After flow cytometric sorting, CD142+ Aregs cells and CD142− non-Aregs cells from ASPCs (the ASPCs were not passaged and were defined as passage 0) were directly seeded into the upper chambers and the bottom layer of 24-well transwell plates (Corning, 3413), respectively, at approximately 60% confluence (about 1.2 × 10^5^ cells, passage 1). The Aregs from Group IV and its corresponding control group (KO ctrl) in the upper chambers were then transferred to another 24-well plate for lentiviral infection. Two days post-infection, an additional 2-day selection with puromycin (1 μg/mL) was performed until 90% of the control uninfected cells had died. Subsequently, the selected Aregs together with the upper chamber were moved back to the original plate containing non-Aregs in bottom layer for continued co-culture (day 0). Meanwhile, on day 0, 6 μg/mL of anti-IGFBP3 antibody (Proteintech, 10189-2-AP) was added to the co-culture medium of Group V. Two days later (day 2), when cell confluence reached 100%, either the apoptosis of non-Aregs in the bottom layer of plates was assessed, or they were subjected to beige differentiation for subsequent experiments.

### 2.12 Annexin V/propidium iodide (PI) apoptosis assay

After 48 h of transwell co-culture, ASPCs from each group were digested with 0.25% trypsin (EDTA-free) and stained using the Annexin V/PI Apoptosis Detection Kit (BD Biosciences, AB_2869082). Briefly, ASPCs were washed with cold PBS and resuspended in 100 μL of 1 × binding buffer at a concentration of approximately 1 × 10^6^ cells/mL. Annexin V-FITC (5 μL) and PI (5 μL) were added, followed by 15 min of incubation at RT in the dark. After incubation, 400 μL of 1 × binding buffer was added to each tube, and apoptosis was immediately analyzed using flow cytometry (Beckman Coulter).

### 2.13 RNA velocity analysis

To investigate cellular dynamics and lineage evolution, RNA velocity analysis was performed on the scRNA-seq data. Unspliced and spliced mRNA transcripts were quantified using velocyto (version 0.17.17) [25]. Subsequently, scVelo (version 0.2.5) was used to identify highly variable genes and conduct dynamic modeling. Loom files were generated from BAM files in the CellRanger output directory using the Velocyto Run10× function. Data preprocessing was conducted with Scv.pp.filter_and_Normalize, followed by RNA velocity estimation using Scv.pp.moments and Scv.tl.velocity in “stochastic mode”. RNA velocity results were integrated with UMAP dimensionality reduction to predict the direction and rate of differentiation from progenitor cells to terminally differentiated cells.

### 2.14 snRNA-seq batch correction, dimensionality reduction, and clustering

The UMI count matrix was imported into Seurat (version 4.4.0) for further analysis [26]. Batch correction across multiple samples was performed using Canonical Correlation Analysis (CCA). Key steps included: (1) log-normalization of the UMI count matrix using NormalizeData() (scale.factor = 10,000) to mitigate differences in total gene expression levels between cells; (2) identification of highly variable genes with FindVariableFeatures() (nFeatures = 2000); (3) identification of integration anchors using FindIntegrationAnchors(); and (4) data integration using IntegrateData() with 30 principal components (nPCs = 30). Cellular subpopulations were identified by applying FindNeighbors() and FindClusters() (resolution = 0.4). Finally, UMAP visualization was performed using RunUMAP() based on the top 15 adjusted CCA dimensions.

### 2.15 Differential expression analysis and cell type annotation

After filtering, a total of 58,714 single cells were grouped into 13 distinct clusters. Differentially expressed genes (DEGs) were identified using the FindAllMarkers() function with parameters “logfc.threshold = 0.25” and “min.pct = 0.1.” P-values were adjusted using the Bonferroni correction method, with a significance threshold of 0.05.

### 2.16 Gene Ontology (GO) enrichment and Kyoto Encyclopedia of Genes and Genomes (KEGG) analysis

Gene sets were annotated using GO terms and KEGG pathways. Enrichment analyses utilized the clusterProfiler R package (version 4.6.2) with the enrichGO and enrichKEGG functions. Annotation was condudted with the org.Mm.eg.db package, and multiple testing corrections were applied using the Benjamini-Hochberg (BH) method (OrgDb = org.Mm.eg.db, pAdjustMethod = “BH”, pvalueCutoff = 0.05). GO terms and KEGG pathways with adjusted p-values (p-adjust) < 0.05 were considered significantly enriched, reflecting their biological relevance to the dataset.

### 2.17 Transcription factor (TF) analysis

TF enrichment and regulon activity were evaluated using the pySCENIC package (version 0.12.0) in Python (version 3.9.20). Gene regulatory networks were constructed based on co-expression and DNA motif analysis. The gene-motif ranking, spanning 10 kb on either side of the transcription start site, was used to delineate the search space for TF regulatory networks. The SCENIC workflow was implemented with default parameters, using the raw count matrix from all samples as input. The analysis consisted of three steps: First, co-expression modules were computed, and relationships between TFs and target genes were weighted with GRNBoost. Second, RcisTarget was applied to identify transcription factors with direct targets (regulons). Finally, AUCell assessed regulon activity in individual cells, and heatmaps visualized the activity of regulons using ggheatmap.

### 2.18 Gene set enrichment analysis (GSEA)

GSEA was conducted on gene expression data from different cell types using the RunGSEA() function in the SCP package (version 0.5.6) to explore biological functions and regulatory mechanisms. The GO Biological Process (GO_BP) database was used for gene set annotation, with Mus musculus as the species reference. Cells were grouped by cell type using the parameter group_by = “CellType”, and only DEGs with adjusted p-values (p_val_adj <0.05) were included for enrichment analysis. Gene set sizes were restricted to a minimum of 10 and a maximum of 500 (minGSSize = 10, maxGSSize = 500), and normalized enrichment scores (scoreType = “std”) were computed. To enhance computational efficiency, parallel processing was enabled using the default configuration of the BiocParallel package.

### 2.19 Inference and analysis of intercellular communication networks

To accurately explore cell–cell interactions within the dataset, three cell communication analysis methods were applied in this study. First, CellChat (version 2.1.2) was used to analyze the differences in intercellular communication between young and aged mice under TN conditions and after 3 or 14 days of cold stimulation. Cell–cell communication probabilities were calculated using the computeCommunProb function (parameter: type = “triMean”) and network centrality scores were derived using the netAnalysis_computeCentrality function (parameter: slot.name = “netP”) to quantify the roles and importance of different cell types within the signaling network. Next, CellPhoneDB was run by converting rat gene names to their human homologs using the homologene tool. The analysis was conducted in a Linux environment, executing CellPhoneDB scripts to generate key output files, including ligand-receptor pair communication significance (pvalues.txt) and average expression levels (means.txt). Visualization was performed using ktplots (version 2.4.0), where heatmaps were created to display the significance and expression levels of ligand-receptor interactions across different cell types. A communication network was then constructed to uncover the signaling patterns between cell types, with a focus on the key interactions between Aregs and SMC-like cells. Lastly, NicheNet (version 2.2.0) was employed to further infer ligand-receptor and ligand–target relationships between Aregs and SMC-like cells. Using the nichenet_seuratobj_aggregate function, Seurat objects were input, with Aregs designated as the signaling sender and SMC-like cells as the receiver. The average expression and standard deviation of ligands were calculated to identify Aregs-specific ligands, from which a ligand-target gene network from Aregs to SMC-like cells was built. A filtering threshold was set at the 40th percentile of the ligand-target gene pair weight distribution, selecting pairs with higher weights. Finally, a Circos plot was used to visualize the interactions between Aregs-specific ligands and their corresponding target genes and receptors in SMC-like cells.

### 2.20 Virtual KO of key genes

In this study, the scTenifoldKnk method [27] was used to perform virtual KO analysis on scRNA-seq data to explore changes in gene regulatory networks and their functional impacts. First, the gene expression matrix was extracted from the scRNA-seq data, and low-expression or noise genes (such as ribosomal genes and mitochondrial genes) were filtered out. Only genes expressed in at least 10 cells were retained to ensure data quality. A wild-type gene regulatory network (WT GRN) was constructed based on scTenifoldKnk, and a KO gene regulatory network (KO GRN) was generated by virtually knocking out the target genes. The KO GRN was then compared to the WT GRN using manifold alignment methods to identify differentially regulated genes (DRGs) associated with the KO of the target gene. DRGs with a significance level meeting the threshold (FDR-adjusted p-value <0.05) were selected for downstream functional analysis. Functional enrichment and pathway analysis of the DRGs were conducted using the STRING protein interaction database and the Enrichr tool to reveal the potential biological significance of the differentially regulated genes. Various visualization methods, including Rank plots, GSEA enrichment plots, and network analysis diagrams, were used to comprehensively display the impact of gene KO on the regulatory network and its potential biological functions.

### 2.21 Statistical analysis

Data are expressed as mean ± SD. Statistical analysis was conducted using one-way ANOVA with GraphPad Prism 9. Statistical significance is indicated as: ∗p < 0.05, ∗∗p < 0.01, ∗∗∗p < 0.001, ∗∗∗∗p < 0.0001.

## 3 Results

### 3.1 Aging leads to an increase in Aregs cells and a decrease in SMC-like cells in murine sWAT following cold exposure

**Figure 1:**
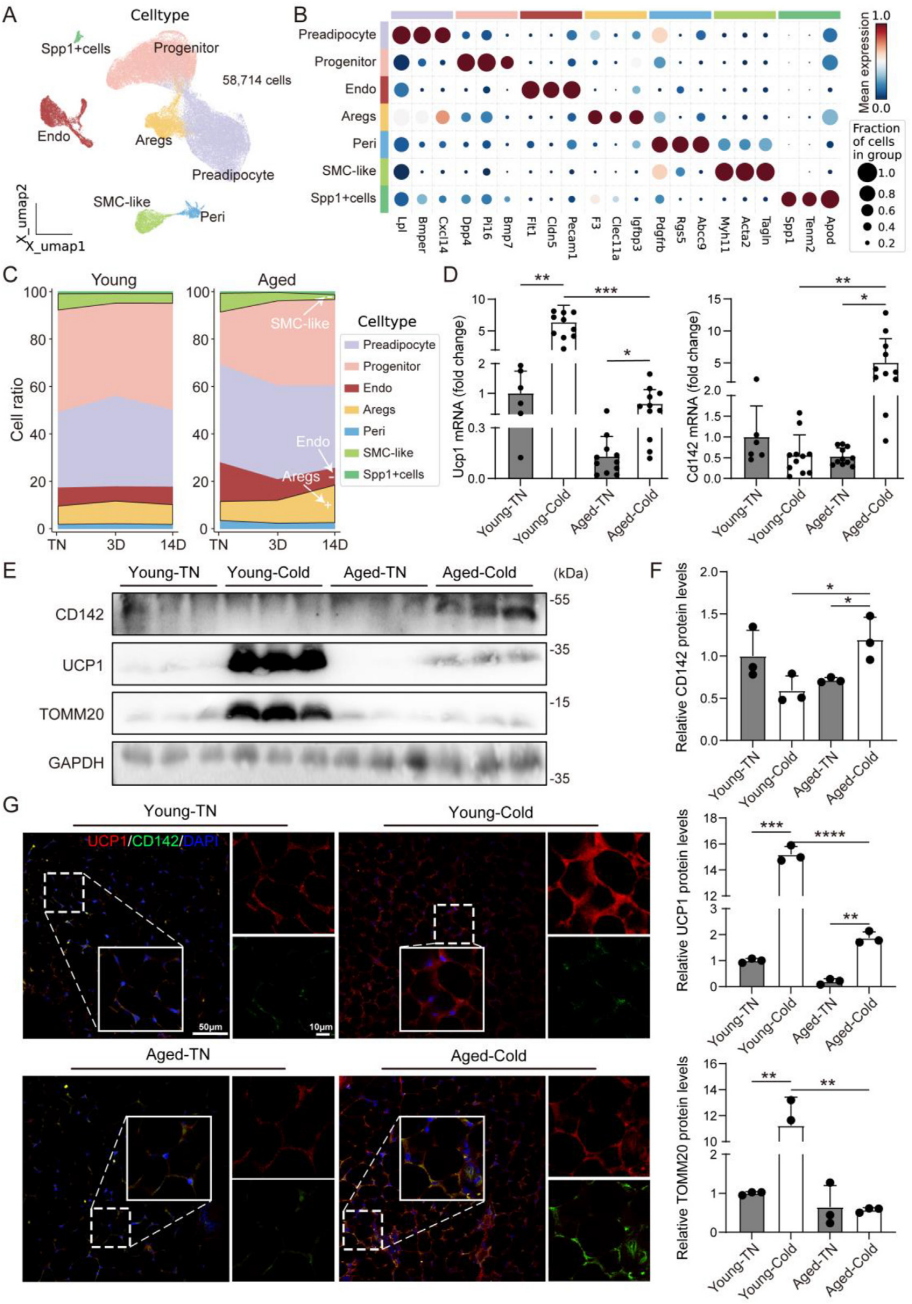
**Aging induces an elevation in Aregs and a reduction in SMC-like cells among ASPCs following cold exposure**. (A) UMAP plot showing the clustering distribution of 58,714 cells from young and aged mice sWAT. (B) Dot plot displaying the expression levels and proportions of selected marker genes across different cell types. The size of the dot represents the percentage of cells expressing the gene, while the color intensity reflects the average gene expression level. (C) Stacked bar chart showing the proportion of cell types in sWAT at different time points (TN, 3 days, and 14 days post-cold exposure). (D) Ucp1 and Cd142 mRNA expression levels analyzed by qPCR in sWAT (n = 11). (E) Immunoblots for CD142, UCP1, TOMM20 and GAPDH in sWAT (n = 3). (F) Quantification of immunoblots of CD142, UCP1 and TOMM20, protein levels are normalized to GAPDH. (G) Immunohistochemistry fluorescence for UCP1 (red) and CD142 (green) (scale bar = 50 μm or 10 μm). Data represent mean ± SD, ∗p < 0.05, ∗∗p < 0.01, ∗∗∗p < 0.001, ∗∗∗∗p < 0.0001.

To assess how aging affects sWAT browning, we integrated scRNA-seq data (GEO: GSE227439) of sWAT from young (9-week-old) and old (57-week-old) mice under different environmental temperatures [thermoneutral (30 °C, TN) and cold conditions (6 °C, Cold)]. After excluding doublets and residual immune cells, a total of 58,714 cells were obtained (29,390 cells from young mice and 29,324 cells from old mice) for downstream analysis. After unbiased clustering using UMAP, we classified all cells into 7 cell types based on the expression of specific marker genes in each cluster. These included five adipose progenitor cell types: Progenitor (Dpp4+), Aregs (Cd142+), Preadipocyte (Cxcl14+), SMC-like cells (Acta2+), and Spp1+ cells. Additionally, two vascular-related cell types were identified: Pericytes (Pdgfrb+) and Endothelial cells (ECs, Flt1+) (Figure 1A,B).

Our analysis revealed changes in the cell composition of sWAT at different time points following cold exposure in mice of different ages. The results showed that in aged mice, the number of Aregs increased with prolonged cold exposure (14D) (Figure 1C and Supplementary Fig. S1), whereas the number of beige adipocyte-specific precursor cells [[3], [4], [5]], SMC-like cells, decreased (14D) (Figure 1C). Notably, this phenomenon was almost absent in young mice. Next, to validate the results of bioinformatics analysis, we first used qPCR to assess the mRNA expression level of the beige adipocyte marker gene Ucp1, confirming the successful establishment of our cold stimulation mouse model. Subsequently, we evaluated the level of Aregs by analyzing the mRNA expression of Cd142. The results showed a significant increase in the Ucp1 mRNA in young mice following cold exposure (14D), whereas the increase in aged mice was less pronounced and remained at lower levels. The result is consistent with numerous previous well-established studies of aging and browning in mice. More importantly, qPCR results also validated a significant upregulation of Cd142 in the cold-exposed aged group (14D) (Figure 1D). Western blot analysis further supported these findings, showing increased expression of UCP1 and the mitochondrial outer membrane protein TOMM20 (used to indicate mitochondrial content) in the cold-exposed sWAT, confirming the successful induction of browning. Additionally, CD142 expression at the protein level was also significantly higher in the cold-exposed aged group (Figure 1E). Similarly, immunohistochemical fluorescence results further corroborated these findings (Figure 1F).

**Figure 2:**
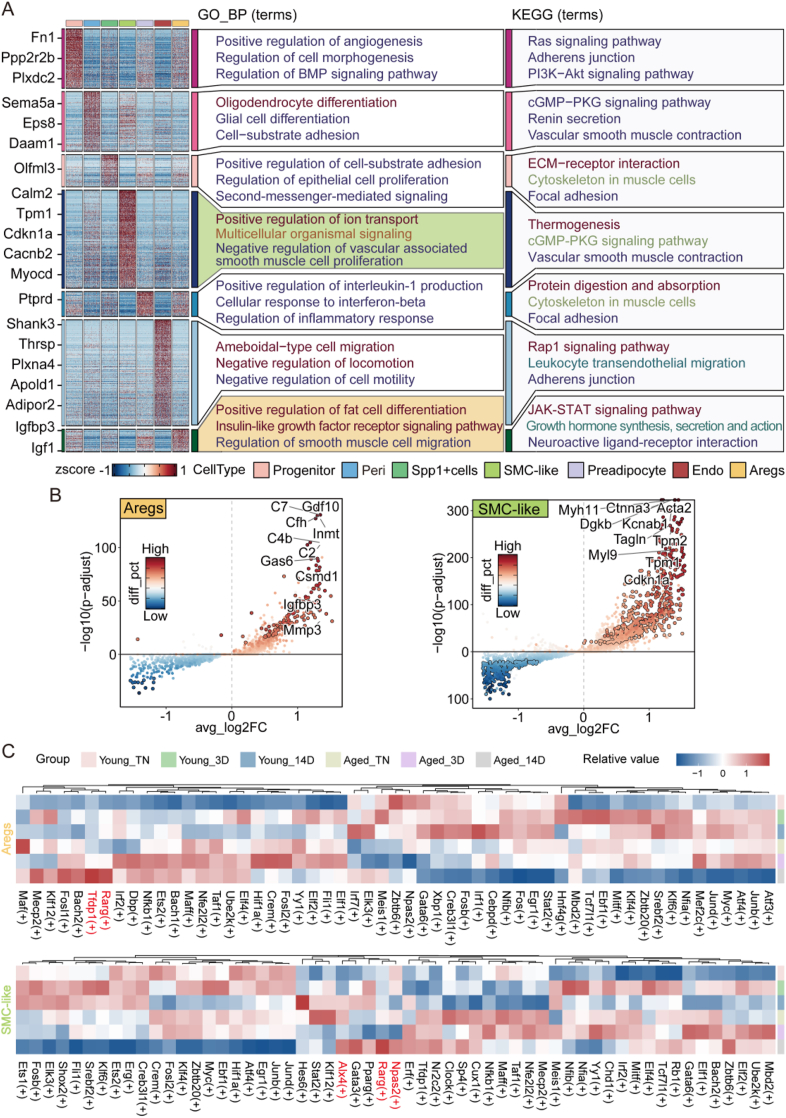
**Analysis of functional properties and molecular characteristics of ASPCs under aged-cold stimulation.** (A) Functional enrichment analysis of different cell types. Left: Heatmap of DEGs for each cell type, showing some DEGs contributing to enrichment. Middle: GO biological processes. Right: KEGG pathways. (B) Volcano plot showing the top 10 upregulated DEGs in Aregs and SMC-like cells. (C) Heatmap of transcription factor analysis for Aregs and SMC-like cells, with colors representing the relative Z-scores of the corresponding transcription factors (red indicating higher reliability and blue representing lower reliability). “+” indicates upregulation.

We performed GO and KEGG pathway analyses for each cell type, revealing that the functions of Aregs were predominantly enriched in “Positive regulation of fat cell differentiation,” “Insulin-like growth factor receptor signaling pathway,” and “Regulation of smooth muscle cell migration.” In contrast, SMC-like cells were enriched in “Positive regulation of ion transport,” “Multicellular organismal signaling,” and “Negative regulation of vascular-associated smooth muscle cell proliferation” (Figure 2A). The pathways enriched in Aregs and SMC-like cells suggest a potential complementary or corresponding relationship between these two cell types in sWAT, highlighting a coordinated regulatory network among different cell subpopulations to enhance adaptability. Further investigation into their interactions may reveal new therapeutic targets for metabolic and vascular diseases.

Subsequently, we performed DEGs analysis on the two significantly altered cell populations, Aregs and SMC-like cells, and visualized the DEGs using volcano plots. Among the top 10 upregulated genes, we identified several noteworthy candidates that, aside from the marker genes, have been rarely emphasized in prior studies but may play roles in the aging-associated impairment of sWAT browning. For instance, Aregs exhibited upregulation of Igfbp3 and complement-related genes (C7, C2, C4b), while SMC-like cells showed increased expression of cell cycle regulatory genes such as Cdkn1a and cytoskeletal genes (Tpm1, Tpm2) (Figure 2B). Studies have shown that complement activation induces inflammation in adipose tissue [28], which negatively impacts the browning process of WAT. The cytoskeleton is essential for maintaining low inflammation levels, regulating proliferation and migration, and ensuring the normal functions of smooth muscle cells [[29], [30], [31]]. This undoubtedly plays a significant role in the browning of WAT. To further reveal the unique transcriptional features of each cell type, we analyzed transcription factors with high transcriptional activity in each cell type (Supplementary Fig. S2), particularly in Aregs and SMC-like cells (Figure 2C). In Aregs (14D), the transcription factor Tfdp1 was highly expressed, with the most significant differential expression observed (encoding PD1), playing an important role in promoting cell apoptosis [32]. Additionally, we also observed high expression of Rarg (encoding retinoic acid receptor) in Aregs (14D) and SMC-like cells (14D), suggesting that Aregs in the sWAT of aged individuals under cold stimulation may further suppress the browning process by inhibiting adipogenesis, potentially leading to the reduced browning capacity of aged adipose tissue. This finding further extends previous research [19]. Moreover, in SMC-like cells (14D), we also observed the expression of pro-apoptotic genes, such as Alx4 and Npas2, which are frequently reported in cancer research [33,34], suggesting they may have an adverse impact on adipose tissue browning. In summary, under aging cold exposure conditions, distinct cell types of ASPCs, especially Aregs and the beige adipocyte precursor SMC-like cells, exhibit specific changes in cell numbers, gene expression, and functional enrichment. These changes may collectively impact the plasticity of adipose progenitor cells and their browning capacity, providing new insights into the imbalance of energy metabolism in aging tissues.

### 3.2 Aregs regulate surrounding ASPCs by IGFBP3-mediated apoptotic signaling

**Figure 3:**
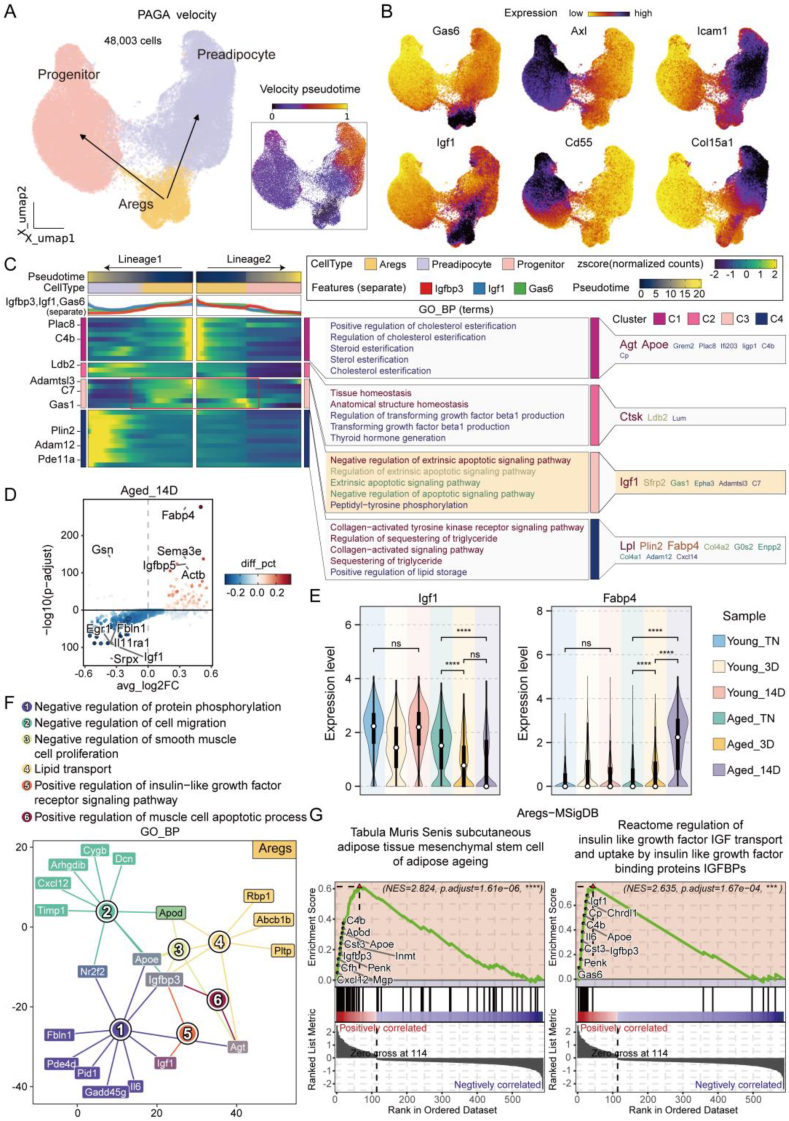
**Aregs influences adjacent ASPCs via IGFBP3-mediated apoptosis signaling.** (A) RNA velocity analysis. Left: PAGA cell dynamics analysis displays differentiation trajectories of three ASPCs subtypes on the UMAP plot. Right: Velocity pseudotime analysis indicates the differentiation extent of the three ASPCs subtypes. (B) UMAP showing the expression distribution of unique genes across the three ASPCs subtypes, with darker colors indicating higher expression levels. (C) Pseudotime analysis of ASPCs. Left: Dynamics of key transition genes along differentiation pathways. Middle: Gene Ontology (GO, biological process). Right: Key enriched genes in the matched pathways. (D) Differential expression analysis highlights the top 5 upregulated and downregulated genes in Aregs cells of aged mice after 14 days of cold stimulation. (E) Violin plots display the expression levels of Igf1 and Fabp4 in Aregs cells under various cold stimulation conditions. (F) Network diagram illustrating the functional enrichment analysis and pathway-related genes specific to upregulated genes in Aregs cells. (G) GSEA analysis based on the MSigDB database shows pathway enrichment for Aregs-specific gene sets. Data represent mean ± SD, ns represents p > 0.05, ∗∗∗∗p < 0.0001.

In our previous results, we identified Aregs as one of the two cell types significantly affected by aging in response to cold stimulation, with potential involvement in regulating aging-associated browning processes. To further elucidate the regulatory capacity of Aregs on surrounding cells, we conducted secondary clustering analysis on 48,003 single cells, including Aregs and two closely related cell types, Progenitor and Preadipocyte. Using Partition-based graph abstraction (PAGA), we performed cell dynamics analysis, which revealed that Aregs, as intermediate-state cells, are likely to regulate the other two cell types. Velocity pseudotime analysis showed that Preadipocytes exhibit a significantly higher degree of differentiation compared to Aregs and Progenitors (Figure 3A), aligning with previous understandings of these cell types [18,35]. Next, we visualized the major upregulated and specifically expressed genes, excluding marker genes, for the three cell types on a UMAP plot. These genes provide molecular evidence for understanding the functions and interconnections of these cells. Among them, Aregs specifically expressed high levels of Gas6 and Igf1, Progenitors showed high expression of Axl and Cd55, while Preadipocytes exhibited specific upregulation of Icam1 and Col15a1 (Figure 3B). Gas6 and Axl, as a ligand-receptor pair, play roles in promoting the activation of BAT, WAT browning [36], and the reduction of inflammation through the promotion of macrophage M2 polarization [37]. The increased expression of Icam1 and Col15a1 in Preadipocytes represents a rise in adhesion molecule and collagen fiber expression during the differentiation of adipocytes toward mature white adipocytes. This change significantly enhances the adhesive capacity of adipocytes, providing a more stable and favorable extracellular matrix environment for their growth and maturation [35]. To further analyze the regulatory capacity of Aregs on surrounding cells, we focused on transition-state genes between different cell types. Pseudotime analysis, with Aregs as the starting cell state, revealed transition-state genes such as Igf1, Sfrp2, Gas1, and Epha3. The GO functional enrichment analysis of these genes showed significant enrichment in pathways such as “regulation of apoptotic signaling pathway” (Figure 3C). Combining these findings with the earlier results and the role of IGFs-mediated apoptotic signaling [[21], [22], [23],^38^], we hypothesize that Aregs play a critical role in modulating the microenvironment between different ASPCs subtypes through the IGFBP3-IGFs signaling-apoptotic pathway.

Given the significant increase in the number of Aregs in aged mice after 14 days of cold stimulation, we specifically examined the DEGs in Aregs under these conditions. Notable upregulated genes included Fabp4, Sema3e, Igfbp5, Actb, and Gsn, while downregulated genes included Igf1, Egr1, Fbln1, Il11ra1, and Srpx (Figure 3D). We observed that the expression of Igf1 was significantly reduced, and Fabp4 was significantly increased in aged mouse Aregs after 14 days of cold stimulation, a phenomenon that was exclusive to aged mice (Figure 3E). Further exploration of the biological processes enriched for these DEGs revealed pathways such as “Negative regulation of protein phosphorylation”, “Negative regulation of cell migration”, “Negative regulation of smooth muscle cell proliferation”, “Positive regulation of muscle cell apoptotic process”, “Lipid transport” and “Positive regulation of insulin-like growth factor receptor signaling pathway”. These findings suggest that Aregs play a crucial role in negatively regulating cell proliferation and migration, with Igfbp3 identified as a central factor linking these pathways (Figure 3F). GSEA analysis using the MSigDB database showed that the gene expression profile of Aregs was significantly enriched in gene sets associated with adipose tissue aging. Enrichment curves highlighted a strong positive correlation for genes such as C4b, Apod, Cst3, Igfbp3 and Cxcl12, indicating that Aregs are closely linked to adipose tissue aging. Additionally, Aregs were highly enriched in Reactome gene sets related to IGFs signaling, with positively correlated cumulative effects for genes including Igf1, Cp, C4b, Igfbp3 and Gas6 (Figure 3G). These results indicate that Aregs, as one of the key cell types responding to aging under cold stimulation, exhibit critical functions in regulating the microenvironment of ASPCs subtypes through Igfbp3 and its modulation of the IGFs-apoptotic pathway.

### 3.3 Apoptotic signaling plays a critical role in SMC-like cells under aged-cold conditions

**Figure 4:**
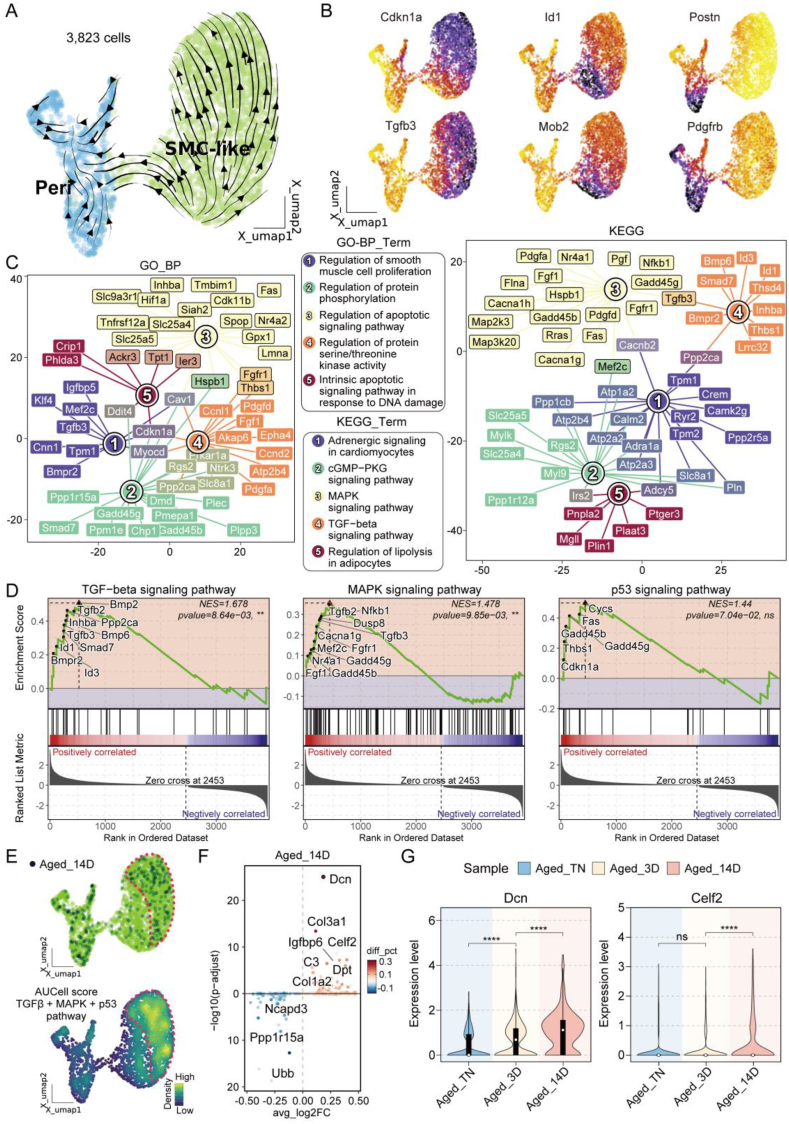
**Apoptotic signaling is pivotal in regulating SMC-like cells under aged-cold conditions.** (A) UMAP integrated with PAGA trajectory analysis illustrating the differentiation trajectories of 3,823 SMC-like and Pericyte cells. (B) UMAP plot showing the expression distribution of specific genes in SMC-like and Pericyte cells, with darker colors indicating higher expression levels. (C) Network diagram depicting GO and KEGG functional enrichment pathways associated with upregulated genes specific to SMC-like cells. (D) GSEA analysis based on the MSigDB database revealing pathway enrichment for SMC-like-specific gene sets. (E) UMAP plot visualizing gene expression of the TGFβ, MAPK, and P53 signaling pathways based on AUCell scoring. (F) Differential expression analysis showing upregulated and downregulated genes in SMC-like cells from aged mice after 14 days of cold stimulation. (G) Violin plot illustrating the expression levels of Dcn and Celf2 in SMC-like cells from aged mice under different cold stimulation conditions. Data represent mean ± SD, ns represents p > 0.05, ∗∗∗∗p < 0.0001.

SMC-like cells, as another cell type with significantly reduced numbers in the aged-cold stimulation group, were analyzed alongside Pericytes, which share a similar expression profile, resulting in a total of 3,823 cells for secondary clustering to refine the analysis of SMC-like cells. UMAP and Velocity trajectory analyses revealed that a subset of less-differentiated SMC-like cells transitions into Pericytes, while another subset progresses toward more differentiated SMC-like cells (Figure 4A). Trajectory gene exploration showed that Pericytes specifically expressed Pdgfrb and Postn, which are consistent with their typical characteristics [39,40]. Meanwhile, SMC-like cells exhibited not only the conventional features but also high expression of Cdkn1a and Tgfb3. TGFβ signaling, which can upregulate Cdkn1a through Smad-dependent mechanisms and is also associated with fibrosis in adipose tissue aging [41,42]. Furthermore, the elevated expression of Cdkn1a suggests its association with the aging and functional exhaustion of SMC-like cells. Additionally, the transition-state genes Id1 and Mob2 are likely to play key roles in the process of SMC-like cell conversion into Pericytes (Figure 4B) (Supplementary Fig. S2).

Subsequently, we performed gene function enrichment analysis on SMC-like cells. GO-BP analysis revealed that SMC-like cells were primarily enriched in pathways such as “regulation of smooth muscle cell proliferation”, “regulation of protein phosphorylation”, “regulation of apoptotic signaling pathway” and “regulation of serine/threonine kinase activity”, suggesting their potential role in maintaining adipose tissue homeostasis and mediating signal transduction. Additionally, KEGG pathway enrichment analysis highlighted key functional modules of SMC-like cells, including “adrenergic signaling in cardiomyocytes”, “cGMP-PKG signaling pathway”, “MAPK signaling pathway”, “TGFβ signaling pathway” and “regulation of lipolysis in adipocytes” (Figure 4C). Based on these initial enrichment findings, we conducted GSEA to evaluate the activation states of signaling pathways (Figure 4E). The analysis demonstrated significant enrichment of the TGFβ signaling pathway (NES = 1.678, p = 8.64e-03). Concurrently, the MAPK signaling pathway also showed significant enrichment (NES = 1.478, p = 9.85e-03), reflecting its critical role in cell proliferation and apoptosis [43]. Although the p53 signaling pathway did not reach statistical significance (NES = 1.44, p = 7.04e-02), the upregulation of key genes such as Cdkn1a, Gadd45b, and Thbs1 suggests its collaborative role with TGFβ and MAPK signaling in inhibiting cell cycling or promoting apoptosis, contributing to the reduction of SMC-like cells. These findings collectively support the hypothesis that apoptotic signaling plays a central regulatory role in the transition and reduction of SMC-like cells (Figure 4D). We further utilized AUCell scoring to project the gene expression patterns of TGFβ, MAPK, and p53 signaling pathways onto the UMAP plot. After 14 days of cold stimulation, a subset of highly differentiated SMC-like cells in aged mice exhibited significant activation of these three pathways (Figure 4E).

To better understand the fate changes of SMC-like cells in the aged-cold group, we performed DEGs analysis on these cells. The results showed that after 14 days of cold stimulation, aged SMC-like cells exhibited upregulation of genes such as Dcn, Col3a1, Igfbp6, Celf2, C3, Dpt, and Col1a2, while genes such as Ubb, Ppp1r15a, and Ncapd3 were downregulated, indicating that these genes may play critical roles in the reduction of SMC-like cells (Figure 4F). The upregulation of fibrosis-related genes (e.g., Dcn, Col3a1, and Col1a2) and inflammation-associated genes (e.g., C3) suggests that cold exposure accelerates the aging and fibrotic processes in these cells. Conversely, the downregulation of genes like Ubb, Ppp1r15a, and Ncapd3, which are involved in protein degradation and cellular stress responses [[44], [45], [46]], further points to the functional exhaustion and senescence of SMC-like cells. Among them, Dcn expression continued to rise significantly as cold stimulation progressed, and Celf2 exhibited a marked increase at the late stage of cold stimulation (Figure 4G). Both DCN and CELF2 have been reported to inhibit cell proliferation and promote cell apoptosis [[47], [48], [49]]. Together, these molecular changes indicate that cold-induced stress exacerbates the reduction of SMC-like cells and contributes to the impaired browning capacity of adipose tissue in aged individuals.

### 3.4 Aregs-SMC-like cells cross-talk regulates apoptosis and impairs beige adipocytes formation

**Figure 5:**
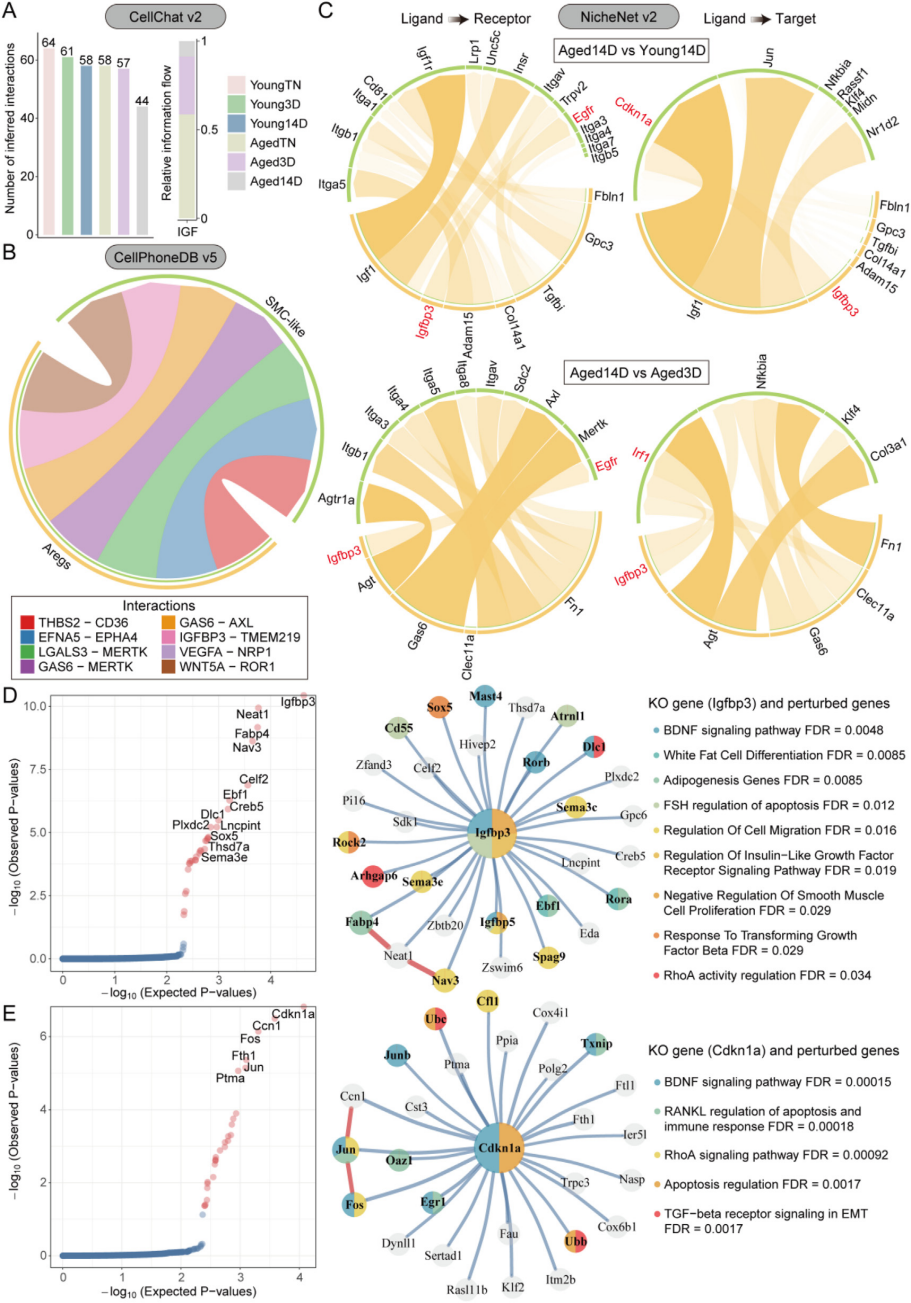
**The cross-talk between aged Aregs and SMC-like cells following cold stimulation.** (A) CellChat: Bar chart showing overall differences in the number of cell–cell interactions following cold stimulation. (B) CellPhoneDB: Chord diagram illustrating apoptosis-related ligand-receptor interactions specific to Aregs and SMC-like cells. (C) NicheNet: Chord diagram displaying ligand-receptor (target) interactions specifically upregulated in the aged 14D group compared to the young 14D group (top) and aged 14D group compared to the aged 3D group (bottom). (D) scTenifoldKnk analysis of Aregs. Left: Rank plot showing 30 perturbed genes influenced by Igfbp3 (p < 0.05); Right: Network diagram depicting the central network and functional enrichment pathways of perturbed genes. (E) scTenifoldKnk analysis of SMC-like cells. Left: Rank plot showing 27 perturbed genes influenced by Cdkn1a (p < 0.05); Right: Network diagram depicting the central network and functional enrichment pathways of perturbed genes.

Considering the opposing trends in cell numbers of Aregs (upregulated) and SMC-like cells (downregulated) under aged-cold stimulation, with Aregs acting as a pro-apoptotic signal donor and SMC-like cells as its recipient, we decided to explore the cross-talk between these two cell types. To systematically elucidate this interaction, we employed three cell communication analysis methods: CellChat v2, NicheNet v2, and CellPhoneDB v5. First, we used CellChat to calculate the number of communications between different cell types. The results revealed that overall intercellular communication was relatively lower in aged mice after 14 days of cold stimulation. Focusing on IGF signaling, which had been highlighted in our previous analyses, we observed that the number of IGF signal-mediated communications progressively decreased with prolonged cold stimulation in the aged group, further corroborating our earlier findings (Figure 5A). Next, we used CellPhoneDB to identify specific ligand-receptor interactions between Aregs (as ligand-producing cells) and SMC-like cells (as recipient cells). This analysis uncovered several unique pro-apoptotic ligand-receptor interactions, such as THBS2-CD36 and IGFBP3-TMEM219 (Figure 5B). Finally, we applied NicheNet to further investigate key ligand-receptor and ligand-target interactions between Aregs and SMC-like cells after 14 days of cold stimulation in aged mice. Strikingly, comparisons between the aged-14 days cold stimulation and the young-14 days cold stimulation group, or between the 14-day and 3-day cold stimulation in aged mice, revealed that Aregs consistently secreted the ligand Igfbp3. This finding underscores the critical role of Igfbp3, which may regulate apoptosis in SMC-like cells by targeting genes such as Cdkn1a and Irf1, as well as the receptor Egfr (Figure 5C). To further explore the functional and regulatory effects of Igfbp3 and Cdkn1a in Aregs and SMC-like cells, respectively, we utilized scTenifoldKnk to perform virtual KO of these genes and examine the resulting gene perturbations within each cell type. In Aregs, the virtual Igfbp3 KO identified Neat1, Fabp4, Nav3, Celf2, and Ebf1 as the top five perturbed genes. Functional enrichment analysis revealed that these perturbed genes were enriched in pathways such as “BDNF signaling pathway”, “white adipocyte differentiation”, “adipogenesis”, “FSH regulation of apoptosis”, “regulation of insulin-like growth factor receptor signaling pathway”, “negative regulation of smooth muscle cell proliferation”, “response to TGFβ” and “RhoA activity regulation” (Figure 5D). Similarly, in SMC-like cells, the virtual KO of Cdkn1a identified Ccn1, Fos, Fth1, Jun, and Ptma as the top five perturbed genes. Functional enrichment analysis indicated that these perturbed genes were associated with pathways such as “BDNF signaling pathway”, “RANKL regulation of apoptosis”, “immune response”, “RhoA signaling pathway”, “regulation of apoptosis” and “TGFβ receptor signaling in EMT” (Figure 5E). In summary, our results demonstrate that under aged-cold stimulation, Aregs secrete Igfbp3, which interacts with multiple targets in SMC-like cells, including Cdkn1a, to establish specific intercellular communication. This interaction enhances apoptosis in SMC-like cells through multiple signaling pathways, ultimately impairing beige adipocytes formation.

### 3.5 Aregs and IGFBP3 inhibit beige adipocytes formation by regulating SMC-like cells in murine ASPCs

**Figure 6:**
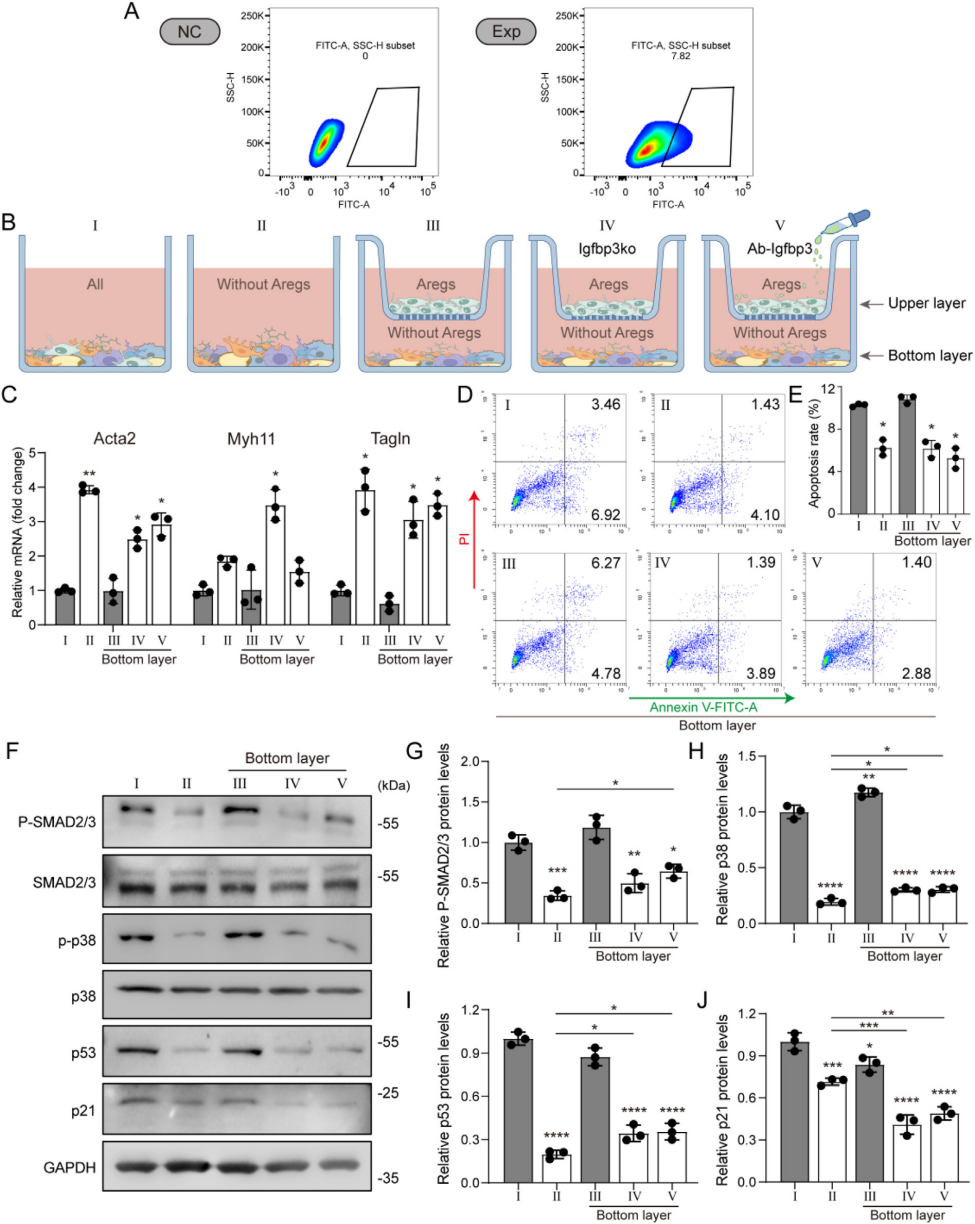
**Aregs and IGFBP3 regulate SMC-like cells apoptosis.** (A–G) Primary ASPCs were isolated and cultured from sWAT of 9-week-old mice and used in subsequent experiments. (A) The flow cytometry sorting results, the left panel represents the negative control group (NC) and the right panel represents the experimental group (Exp). (B) Schematic diagram of transwell co-culture groups (Ab-IGFBP3 was added at a concentration of 6 μg/mL (Supplementary Figs. S6A–D) [50]). (C) Myh11, Acta2 and Tagln mRNA expression levels analyzed by qPCR. (D) Annexin V/PI staining followed by flow cytometry analysis. (E) Proportion of Annexin V+ apoptotic cells. (F) Immunoblots for P-SMAD2/3, SMAD2/3, P-p38, p38, p53, p21 and GAPDH. (G–J) Quantification of P-SMAD2/3 immunoblotting, normalized to SMAD2/3; P-p38 immunoblotting, normalized to p38; and p53 and p21 immunoblotting, normalized to GAPDH. Data represent mean ± SD, ∗p < 0.05, ∗∗p < 0.01, ∗∗∗p < 0.001, ∗∗∗∗p < 0.0001.

To clarify the impact of Aregs and its secreted IGFBP3 on the number of SMC-like cells and the subsequent impairment of beige adipocytes formation, we performed flow cytometric sorting of primary ASPCs isolated from murine sWAT to separate CD142+ (Aregs) and CD142− (non-Aregs), which were then co-cultured in different groups using transwell assays (Figure 6B). Flow sorting results showed that, compared to the negative control group incubated only with FITC-conjugated secondary antibody (Figure 6A left), the experimental group yielded approximately 7.82% CD142+ cells (Figure 6A right), which closely matched the proportion of Aregs cells observed in our previous bioinformatic analysis, confirming successful separation of Aregs cells. To further validate the success of flow sorting, we collected groups I, II, and Aregs for qPCR and immunoblotting. Results showed that CD142 mRNA and protein levels in group II were significantly lower than in group I, while Aregs group showed significantly higher levels compared to group I (Supplementary Figs. S4A, B, C). This further confirmed the successful separation of Aregs cells. Additionally, we verified IGFBP3 KO in the Aregs of group IV using qPCR and immunoblotting. The results showed that, compared to group I and Aregs group, IGFBP3 mRNA and protein levels were significantly lower in IGFBP3 KO of Aregs, confirming successful KO of IGFBP3 (Supplementary Figs. S4D, E, F). Next, we compared the expression levels of SMC-like cell markers (Myh11, Acta2 and Tagln) in the bottom layers of groups I, II, III, IV, and V. Strikingly, qPCR results showed that Acta2 and Tagln mRNA levels were significantly higher in the groups II, bottom layers of IV and V compared to groups I and bottom layers of III, although Myh11 did not show significant differences in all groups (Figure 6C). To further clarify the pro-apoptotic effects of Aregs and their secreted factor IGFBP3 on SMC-like cells, we performed an Annexin V/PI apoptosis assay on ASPCs from each group. Flow cytometry results demonstrated that the apoptosis rates of ASPCs in Group II and the bottom layers of Groups IV and V were significantly lower than those in Group I and the bottom layer of Group III (Figure 6C, D). Meanwhile, to further explore and validate the bioinformatics analysis results, we examined the activation of the TGFβ, MAPK, and p53 pathways in ASPCs from each group. Immunoblotting results revealed that the protein levels of P-SMAD2/3, P-p38, p53, and p21 were suppressed to varying degrees in Group II and the bottom layers of Groups IV and V compared to Group I and the bottom layer of Group III, indicating that these three signaling pathways were inhibited either independently or cooperatively when Aregs or their secretory factor IGFBP3 were removed (Figure 6F–J).

**Figure 7:**
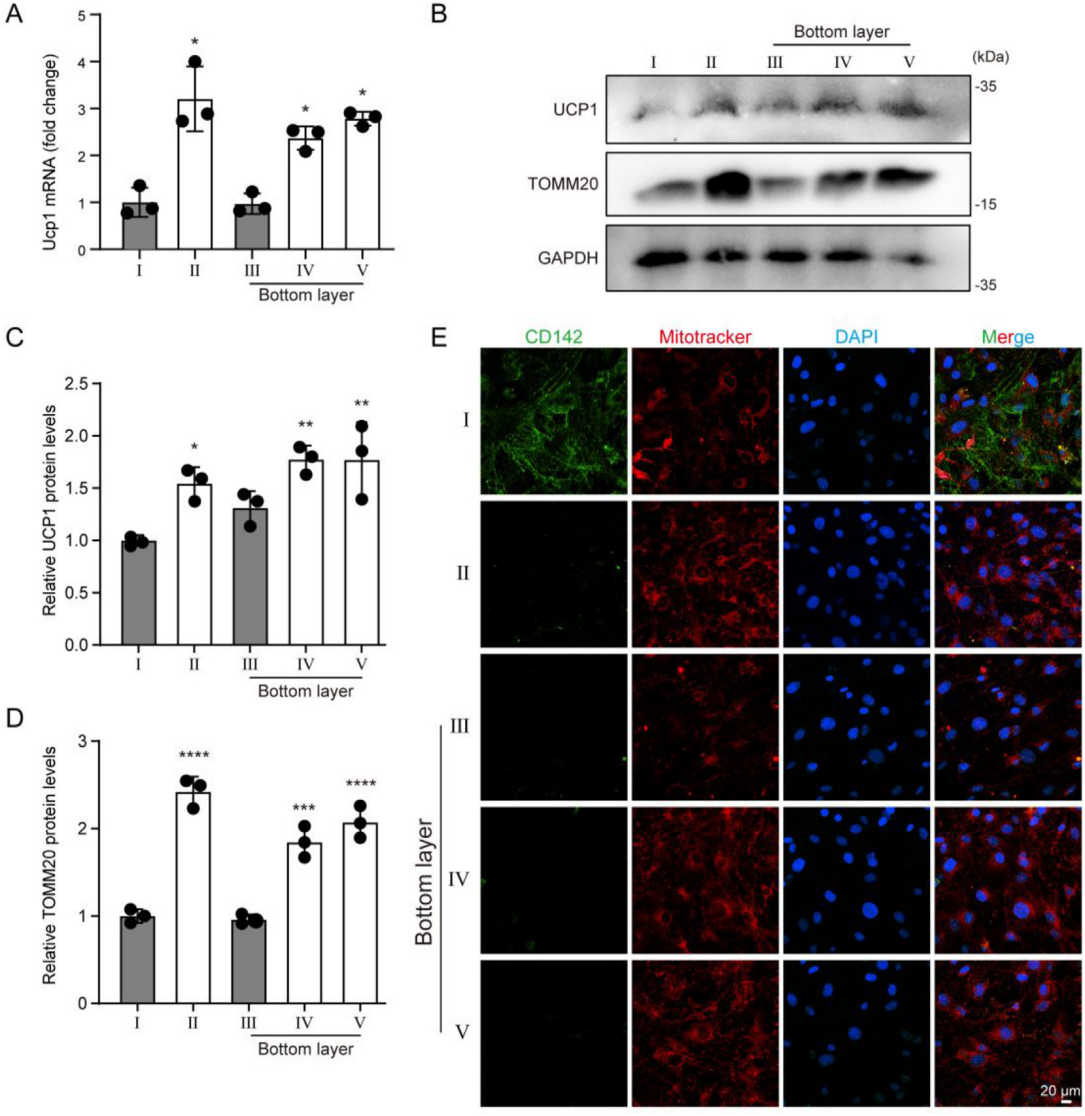
**Aregs and IGFBP3 inhibit beige adipocyte formation.** (A–E) All five groups of ASPCs underwent the same browning differentiation protocol. (A) Ucp1 mRNA expression levels analyzed by qPCR. (B) Immunoblots for UCP1, TOMM20 and GAPDH. (C,D) Quantification of immunoblots of UCP1 and TOMM20, protein levels are normalized to GAPDH. (E) Immunofluorescence staining for CD142 and Mitotracker for mitochondria (scale bar = 20 μm). Data represent mean ± SD, ∗p < 0.05, ∗∗p < 0.01, ∗∗∗p < 0.001, ∗∗∗∗p < 0.0001.

Subsequently, all five groups underwent the same browning differentiation protocol. After differentiation, the results showed that the mRNA and protein levels of the beige adipocytes marker UCP1 in the groups II, bottom layers of IV and V were significantly higher than those in groups I and bottom layers of III (Figure 7A, B, C). Another marker of beige adipocytes, high mitochondrial content, was also observed in the groups II, bottom layers of IV and V (Figure 7B, D, E). In conclusion, our results demonstrate that Aregs and their secreted factor IGFBP3 in murine primary ASPCs play a significant role in inhibiting beige adipocytes formation, which may be a key factor contributing to the impaired sWAT browning capacity of aged mice.

## 4 Discussion

Our study demonstrates the crucial role of Aregs and their secreted factor IGFBP3 in the impaired browning capacity of aged sWAT under cold stimulation. We show that Aregs, which are upregulated in aged mice following cold exposure, secrete IGFBP3, which acts as a pro-apoptotic signal for SMC-like cells. This interaction between Aregs and SMC-like cells leads to apoptosis, impairs the function of SMC-like cells, and ultimately reduces the formation of beige adipocytes.

Adipose tissue serves not only as an energy reservoir but also plays a crucial role in maintaining systemic health through the secretion of adipokines and extracellular vesicles [51,52]. Beige adipocytes, in addition to sharing these functions, promote lipid and glucose metabolism by dissipating excess energy as heat, a process mediated by UCP1. This enhanced metabolic activity helps mitigate or prevent metabolic diseases, including CVD, through mechanisms such as lowering blood triglycerides and non-HDL cholesterol levels, increasing HDL cholesterol levels, accelerating the clearance of triglyceride-rich lipoprotein (TRL) remnants, and secreting cardioprotective adipokines [9,10,53]. These findings highlight the potential therapeutic value of targeting WAT browning in the management of metabolic diseases.

Given the promising therapeutic potential of beige adipose tissue, an increasing number of researchers are exploring factors that can promote browning process from various perspectives, including pharmacological interventions, genetic modifications, and GPCRs [[54], [55], [56], [57], [58]]. However, the inevitable decline in browning capacity observed in aging individuals exacerbates metabolic dysregulation and disrupts homeostasis [11,12,59]. This underscores the importance of understanding the molecular mechanisms behind adipose browning in aging individuals and how these processes can be leveraged for therapeutic interventions. WAT browning occurs through two primary mechanisms: one involves the trans-differentiation of white adipocytes into beige adipocytes, and the other involves the de novo differentiation of beige adipocytes from specialized progenitor cells, SMC-like cells [60]. These SMC-like cells exhibit characteristics of both adipose progenitor cells and smooth muscle-like cells, playing a critical role in the direct formation of beige adipocytes [3,60,61]. In practice, both mechanisms coexist, but the predominance of each varies depending on external conditions. Reports have shown that in mice maintained at ambient temperature (20–23 °C) before cold stimulation, trans-differentiation is predominant, whereas in mice preconditioned at thermoneutrality (30 °C) before cold exposure, de novo differentiation dominates the process [62]. The publicly available scRNA-seq data and the mouse models used in our experiments were derived from TN-to-cold stimulation conditions, which enabled us to focus on the impact of SMC-like cells on browning capacity.

Aregs, as a novel cell subtype in ASPCs, were first reported by Schwalie et al. and have since been primarily studied for their anti-adipogenic effects in white adipocytes [17]. The mechanisms involved mainly focus on the paracrine actions of Aregs, with key genes such as Rtp3, Spink2, and Vitthe, as well as the retinoic acid pathway [17,19]. In our study, we provide the first evidence of an increase in Aregs cell numbers in aged individuals' sWAT under cold stimulation and, for the first time, report their inhibitory role in beige adipocytes formation. Our findings extend this understanding by showing that Aregs secrete IGFBP3 and act as an important mediator in the cross-talk between Aregs and SMC-like cells, emphasizing its potential as a therapeutic target for reversing age-related adipose dysfunction. However, we noted that Holman et al. did not emphasize changes in ASPC subpopulation proportions, including Aregs and SMC-like cells, despite our scRNA-seq raw data being sourced from their study (Holman et al., 2024, eLife) [14]. To understand the origin of these discrepancies, we compared the analytical processes and found that differences in raw data processing, including the Cell Ranger version and reference genome selection, resulted in variations in cell clustering. Additionally, the strict filtering criteria employed by Holman et al. excluded moderately expressed cells, such as those from the aged-cold 14-day group, leading to a reduced number of analyzed cells. In contrast, we applied a two-stage quality control (QC) strategy, allowing us to obtain a larger and more comprehensive dataset. Regarding CD142+ ASPCs, Holman et al. reported normal adipogenesis, while other studies [17,19,63,64], including ours, demonstrate that Aregs inhibit adipogenesis. These discrepancies may stem from differences in experimental protocols. For instance, we exclusively used primary ASPCs isolated from young mice and differentiated them under higher insulin concentrations (850 nM). Moreover, during cell sorting, it is possible that a minor proportion of CD142+ cells with adipogenic potential (CD142+, ICAM1+, and VAP1+), as reported in a previous study [63], were more or less inadvertently included.

IGFBP3 influences the apoptosis of SMC-like cells through IGF-dependent or IGF-independent signaling pathways. Our analysis reveals that SMC-like cells in aged mice exhibit increased expression of apoptosis markers, such as Cdkn1a, and functional enrichment analysis highlights the activation of TGFβ and MAPK signaling pathways, both of which are known to induce apoptosis and fibrosis in adipose tissue [42,43,65,66]. Notably, our results suggest that IGFBP3 secreted by Aregs interacts with receptors and signaling molecules in SMC-like cells, including EGFR and Cdkn1a, thereby enhancing the apoptosis process [[67], [68], [69]]. These findings provide a mechanistic link between Aregs and the reduced thermogenic capacity of aged adipose tissue. Additionally, our co-culture experiments demonstrate that IGFBP3 significantly inhibits the differentiation of beige adipocytes, which is consistent with the reduced expression of SMC-like cell markers (Acta2, Myh11, and Tagln), further confirming its role in the decline of sWAT browning capacity.

It is worth mentioning, in addition to Aregs and SMC-like cells, there is another significantly altered cell subpopulation, ECs. ECs and SMC-like cells are reported to have a mutually regulatory role as the primary cell types in the intima and media of blood vessel walls, respectively, influencing the formation of vascularization [[70], [71], [72], [73], [74]], which is crucial for supporting the development of beige adipose tissue [75,76]. Therefore, we conducted preliminary explorations, and our results indicated that despite a general reduction in ECs, a specific subpopulation of arterial endothelial cells (Mgp + ArtEC) was significantly upregulated in aged mice following cold exposure. Based on our initial findings, we hypothesize that changes in SMC-like cells may lead to a reduction in other endothelial cells through the upregulation of Mgp + ArtEC, ultimately impairing vascularization and affecting the formation of beige adipose tissue in aged mice (Supplementary Fig. S7). This warrants further exploration in future studies.

Although our study provides important insights into the roles of Aregs and SMC-like cells, several limitations remain to be addressed. First, while our research offers valuable understanding of the molecular mechanisms underlying impaired adipose browning during aging and has conducted preliminary explorations of the apoptotic signaling pathways involved, further studies are needed to pinpoint whether the TGFβ, MAPK, and p53 signaling pathways have synergistic, partially synergistic, or independent effects on the apoptosis of SMC-like cells. Second, the use of in vitro co-culture systems may not fully replicate the physiologically complex interactions occurring in vivo. Future studies utilizing lineage tracing models and conditional KO of CD142 or IGFBP3 in specific cell populations, as well as the development of peptides targeting CD142+ cells, will provide more definitive evidence of their role in adipose tissue aging and offer a more accurate understanding of the molecular mechanisms involved, including the signaling pathways. Moreover, the long-term effects of regulating Aregs or IGFBP3 on systemic metabolism and thermogenesis remain to be explored.

In conclusion, our findings elucidate the key role of Aregs and IGFBP3 in mediating the impaired browning capacity of aged sWAT under cold stimulation. By revealing the molecular mechanisms underlying this process, our study lays the foundation for developing therapeutic strategies to combat age-related metabolic dysfunction.

## 5 Conclusion

In this study, we identified the critical role of Aregs and its secreted factor IGFBP3 in regulating the browning process of aged adipose tissue. Our results indicate that Aregs, through IGFBP3-mediated apoptosis of SMC-like cells, significantly impairs the formation of beige adipocytes in aged mice, leading to the loss of thermogenic capacity in aged WAT. These findings provide insights into the molecular mechanisms underlying the reduction in adipose tissue browning with aging, particularly from the perspective of de novo differentiation, and highlight Aregs and IGFBP3 as potential therapeutic targets for preventing or reversing age-related metabolic dysfunctions.

## CRediT authorship contribution statement

**Shifeng Wang:** Writing – original draft, Visualization, Validation, Resources, Methodology, Investigation, Formal analysis, Data curation. **Yuanxu Cui:** Writing – review & editing, Writing – original draft, Visualization, Investigation, Funding acquisition. **Limei Wang:** Writing – review & editing, Investigation, Funding acquisition. **Chun Feng:** Writing – review & editing, Investigation, Funding acquisition. **Yifei Sun:** Methodology, Formal analysis. **Bangyun Huo:** Investigation, Formal analysis. **Honglu Jiang:** Investigation, Formal analysis. **Mingyu Zhao:** Resources, Data curation. **Yingying Tu:** Resources. **Qiyue Wang:** Resources. **Yutao Yang:** Resources. **Qiang Zhang:** Writing – review & editing, Supervision, Resources, Methodology, Investigation, Funding acquisition, Data curation, Conceptualization.

## Funding sources

This research was funded by the Kunming Medical University (China) for High-Level Talent Research Supporting Fund (grant K132310515 to Q.Z. and grant K132310535 to Y.X.C.), Yunnan Provincial Department of Education Scientific Research Fund Project (grant 2025J0287 to Q.Z. and grant 2025J0255 to Y.X.C.), National Natural Science Foundation of China (grant 82460164 to L.M.W.) and The cultivation of the discipline team in Medical Experimental Animal Science at Kunming Medical University (grant 2024XKTDPY17 to Q.Z. and L.M.W.).

## Declaration of competing interest

The authors declare that they have no known competing financial interests or personal relationships that could have appeared to influence the work reported in this paper.

## Data Availability

Data will be made available on request.
