# Supplementary material for: Aregs-IGFBP3-mediated SMC-like cells apoptosis impairs beige adipocytes formation in aged mice
Source: Mol Metab. 2025 Mar 19;95:102125. doi: 10.1016/j.molmet.2025.102125 (PMC11985090; doi:10.1016/j.molmet.2025.102125)
Supplement: Multimedia component 1 [file mmc1.docx]

**Supplementary Information**

**Aregs-IGFBP3-mediated SMC-like cells apoptosis impairs beige adipocytes formation in aged mice**

Shifeng Wang, Yuanxu Cui, Limei Wang, Chun Feng, Yifei Sun, Bangyun Huo, Honglu Jiang, Mingyu Zhao, Yingying Tu, Qiyue Wang, Yutao Yang, Qiang Zhang

Contents:

Supplementary Table 1

**Supplementary Table 1.** Primer sequences.

| Primer name (murine) | Primer sequence |
| --- | --- |
| Cd142 | Forward Primer: 5′-GGGAGGAGCCGCCATTTACAAAC-3′ |
|  | Reverse Primer: 5′-AGACTTGCCGCAGGGTGAGG-3′ |
| Igfbp3 | Forward Primer: 5′-CCGAGTGACCGATTCCAAGTTCC-3′ |
|  | Reverse Primer: 5′-AGTTCTGGGTGTCTGTGCTTTGAG-3′ |
| Ucp1 | Forward Primer: 5′-GAAACACCTGCCTCTCTCGGAAAC-3′ |
|  | Reverse Primer: 5′-GCATTCTGACCTTCACGACCTCTG-3′ |
| Myh11 | Forward Primer: 5′-CAAGATCGTGAAGACCAGTCCATTC-3′ |
|  | Reverse Primer: 5′-AGGATGCCACCACAGCCAAG-3′ |
| Tagln | Forward Primer: 5′-ATGGAACAGGTGGCTCAATTCTTG-3′ |
|  | Reverse Primer: 5′-CTTCATAGAGGTCAACAGTCTGGAAC-3′ |
| Acta2 | Forward Primer: 5′-ATGACCCAGATTATGTTTGAGACCTTC-3′ |
|  | Reverse Primer: 5′-TCTCCAGAGTCCAGCACAATACC-3′ |
| Gapdh | Forward Primer: 5′-ACTCTTCCACCTTCGATGC-3′ |
|  | Reverse Primer: 5′-CCGTATTCATTGTCATACCAGG-3′ |
