# Supplementary material for: Aregs-IGFBP3-mediated SMC-like cells apoptosis impairs beige adipocytes formation in aged mice
Source: Mol Metab. 2025 Mar 19;95:102125. doi: 10.1016/j.molmet.2025.102125 (PMC11985090; doi:10.1016/j.molmet.2025.102125)
Supplement: Multimedia component 2 [file mmc2.docx]

**Supplementary Information**

**Aregs-IGFBP3-mediated SMC-like cells apoptosis impairs beige adipocytes formation in aged mice**

Shifeng Wang, Yuanxu Cui, Limei Wang, Chun Feng, Yifei Sun, Bangyun Huo, Honglu Jiang, Mingyu Zhao, Yingying Tu, Qiyue Wang, Yutao Yang, Qiang Zhang

Contents:

Supplementary Figure S1-S7


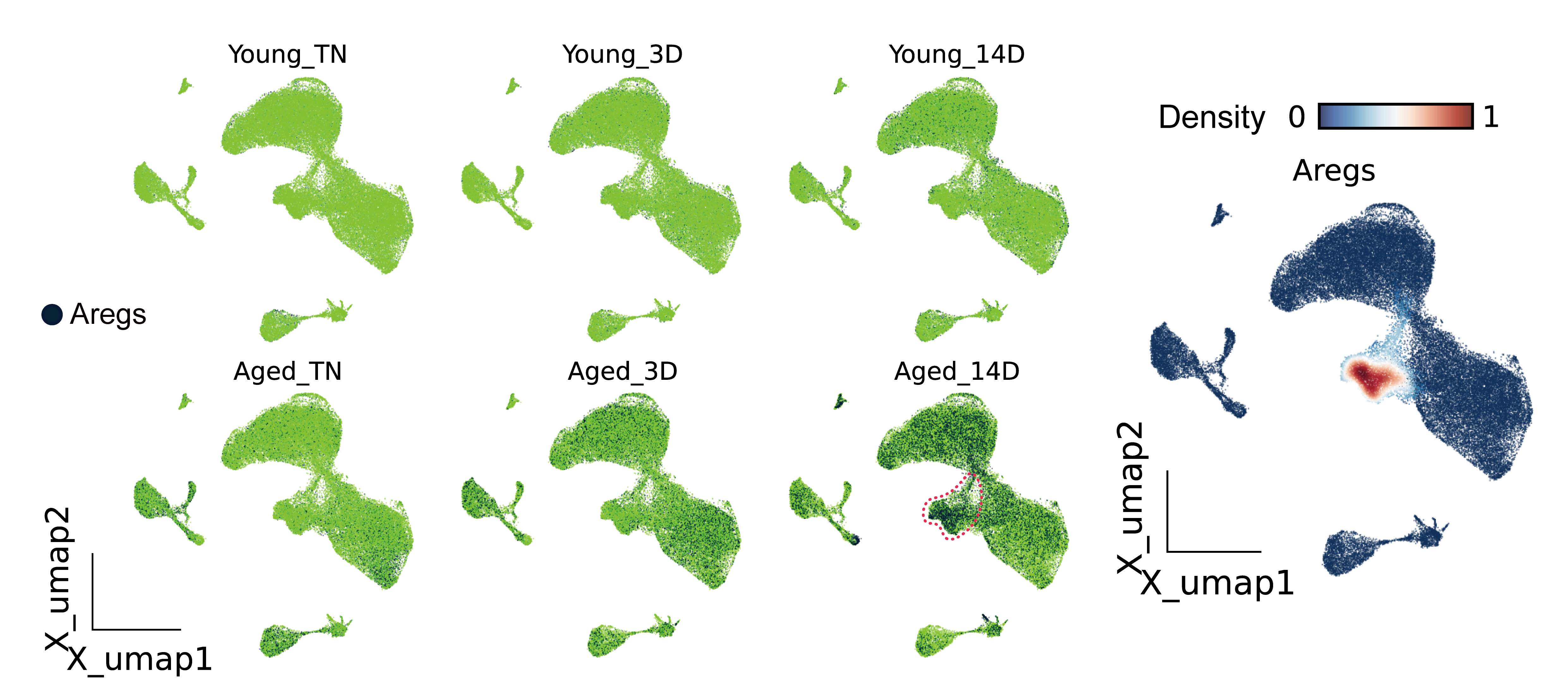


**Supplementary Figure S1:** UMAP projection illustrating the distribution of Aregs cells across different groups, along with the density patterns of Aregs cell distribution.


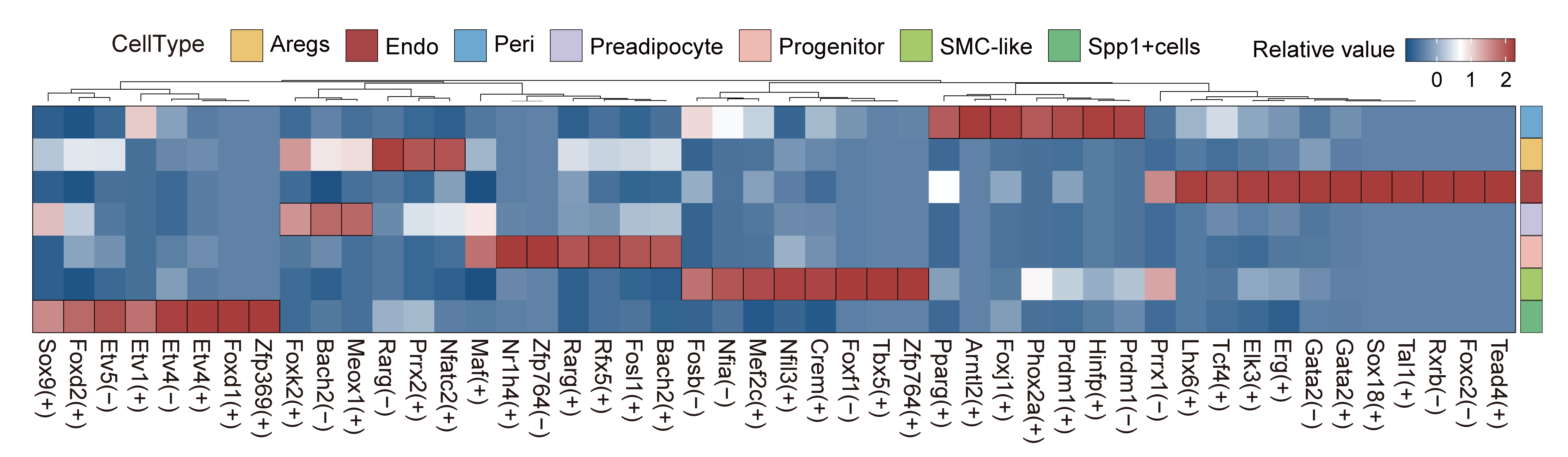


**Supplementary Figure S2:** The heatmap displays cell type-specific transcription factors, with colors representing the relative Z-scores of the corresponding transcription factors (red indicating higher reliability and blue representing lower reliability). "+" indicates upregulation, while "-" indicates downregulation.


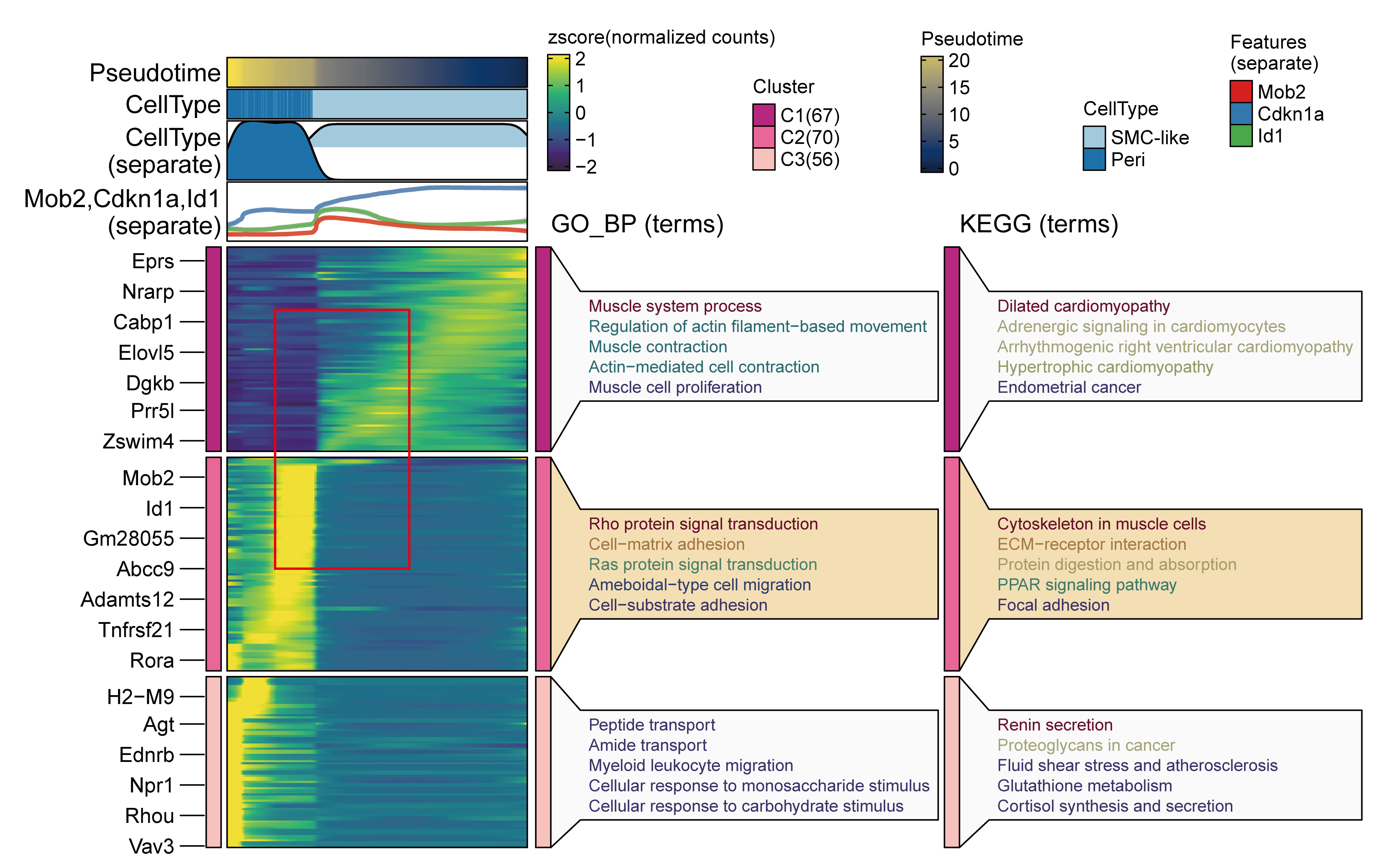


**Supplementary Figure S3:** Pseudotime analysis of SMC-like cells. Left: Dynamic regulation of several key transitional genes along the differentiation trajectory. Middle: GO-BP analysis of temporal genes. Right: KEGG pathway analysis of temporal genes.


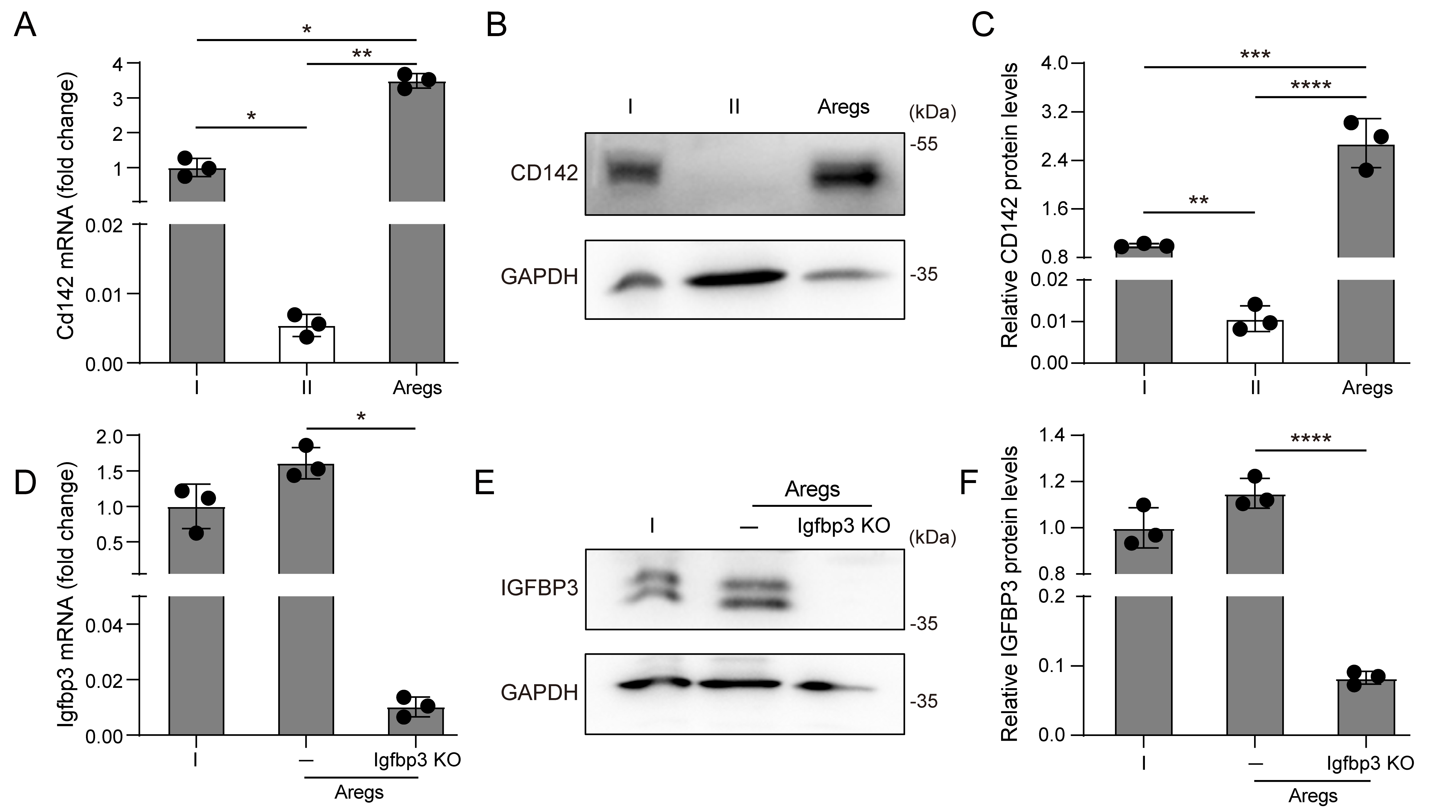


**Supplementary Figure S4:** **Identification of effective sorting of CD142+ and CD142− ASPCs and effective KO of Igfbp3 in Aregs.** (A) Cd142 mRNA expression levels analyzed by qPCR in indicated groups. (B) Immunoblots for CD142 and GAPDH. (C) Quantification of immunoblots of CD142, protein levels are normalized to GAPDH. (D) Igfbp3 mRNA expression levels analyzed by qPCR. (E) Immunoblots for IGFBP3 and GAPDH. (F) Quantification of immunoblots of IGFBP3, protein levels are normalized to GAPDH. Data represent mean ± SD, *p < 0.05, **p < 0.01, ***p < 0.001, ****p < 0.0001.


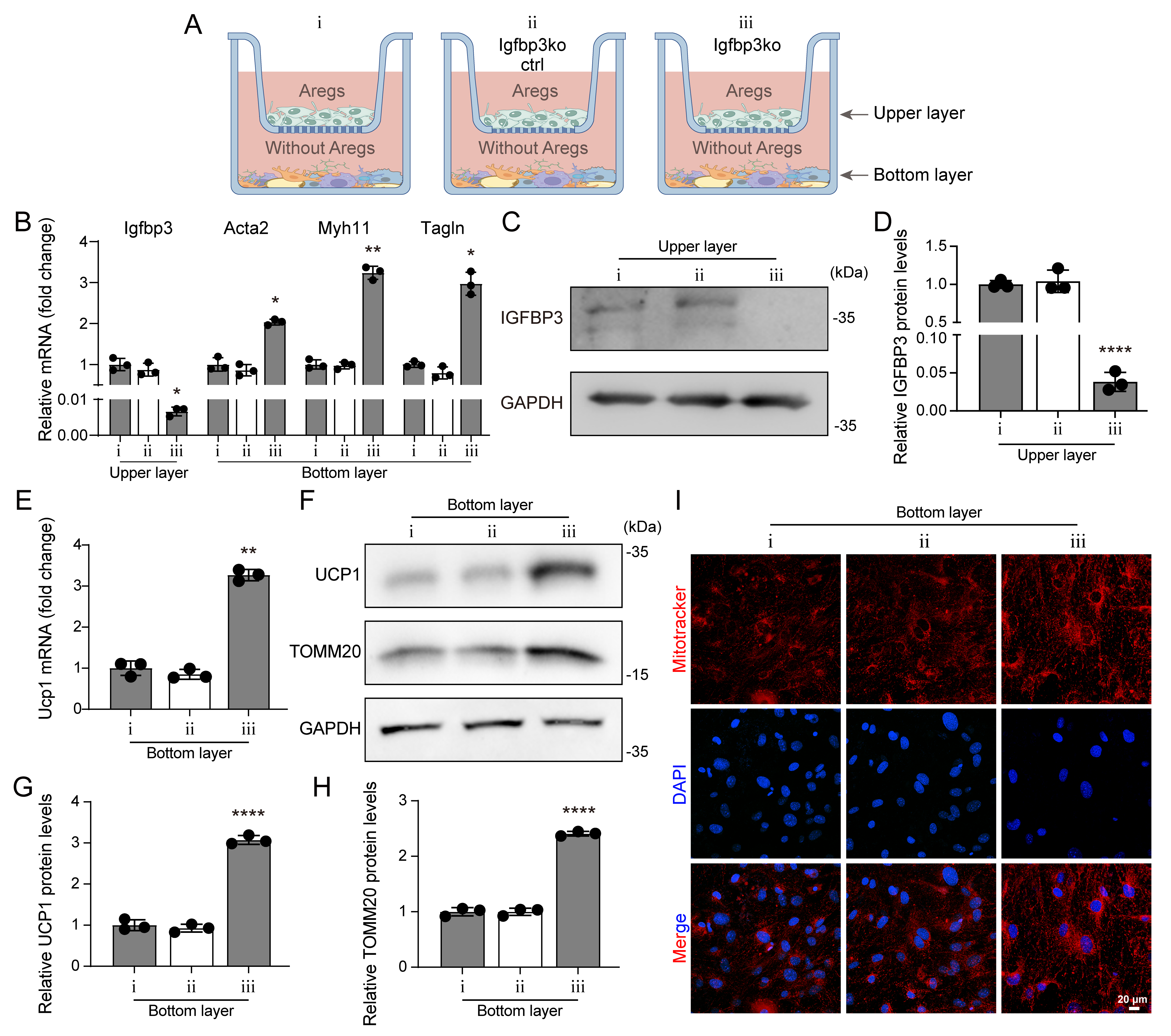


**Supplementary Figure S5:** **Igfbp3 KO ctrl of Aregs does not affect beige adipogenesis in non-Aregs compared to Igfbp3 KO.** (A-D) ASPCs did not undergo differentiation. (A) Schematic diagram of designated transwell co-culture groups. (B) Igfbp3, Acta2, Myh11 and Tagln mRNA expression levels analyzed by qPCR. (C) Immunoblots for IGFBP3 and GAPDH. (D) Quantification of immunoblots of IGFBP3, protein levels are normalized to GAPDH. (E-I) ASPCs underwent beige adipocyte differentiation. (E) Ucp1 mRNA expression levels analyzed by qPCR. (F) Immunoblots for UCP1, TOMM20 and GAPDH. (G,H) Quantification of immunoblots of UCP1 and TOMM20, protein levels are normalized to GAPDH. (I) Fluorescent staining of mitochondria using Mitotracker (scale bar = 20 μm). Data represent mean ± SD, *p < 0.05, **p < 0.01, ***p < 0.001, ****p < 0.0001.


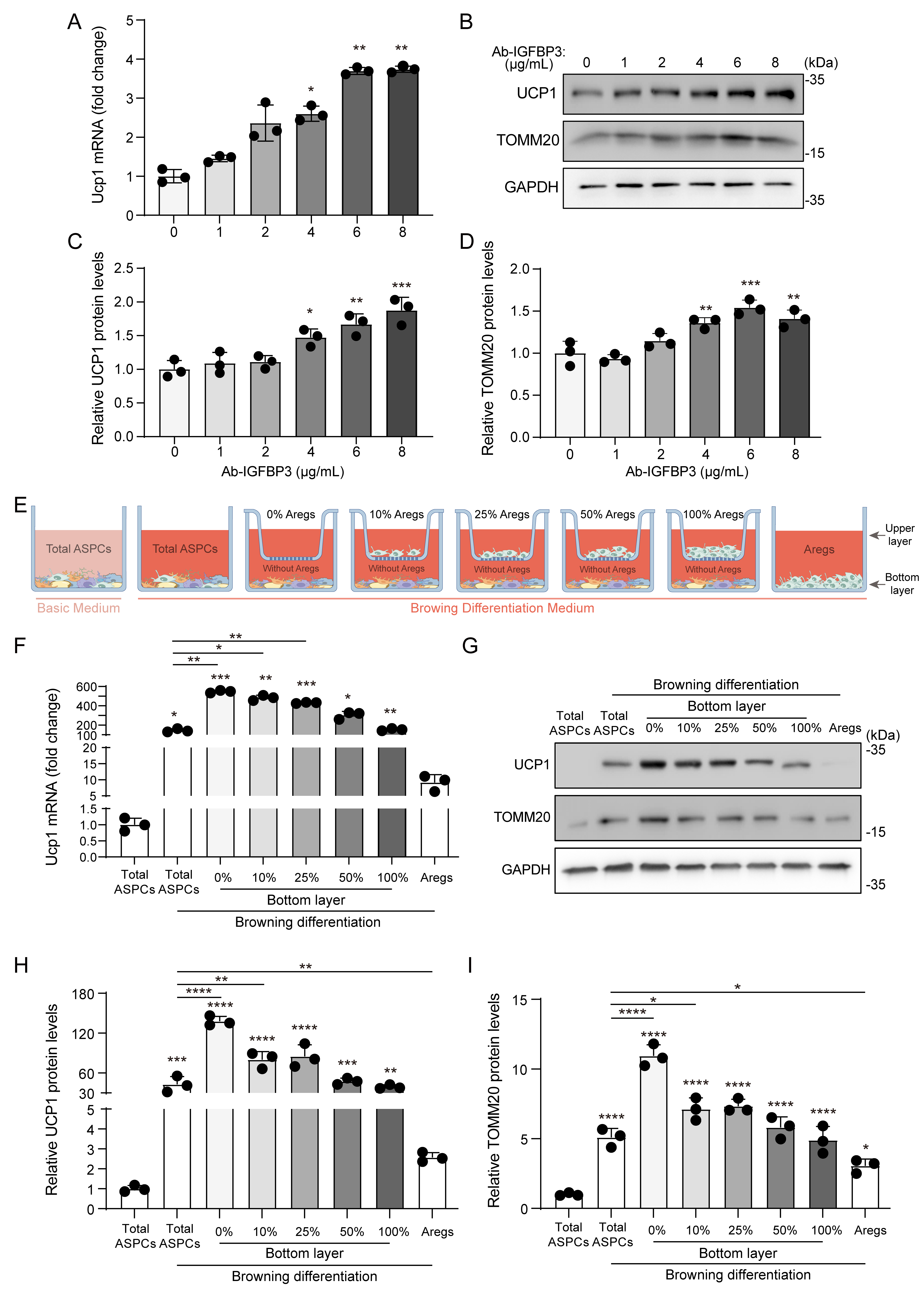


**Supplementary Figure S6:** **Effect of different anti-IGFBP3 antibody concentrations on promoting beige adipogenesis in non-Aregs and the impact of varying Aregs cell numbers on non-Aregs**. (A) Ucp1 mRNA expression levels analyzed by qPCR. (B) Immunoblots for UCP1, TOMM20 and GAPDH. (C,D) Quantification of immunoblots of UCP1 and TOMM20, protein levels are normalized to GAPDH. (E) Schematic diagram of designated transwell co-culture groups. (F) Ucp1 mRNA expression levels analyzed by qPCR. (G) Immunoblots for UCP1, TOMM20 and GAPDH. (H,I) Quantification of immunoblots of UCP1 and TOMM20, protein levels are normalized to GAPDH.


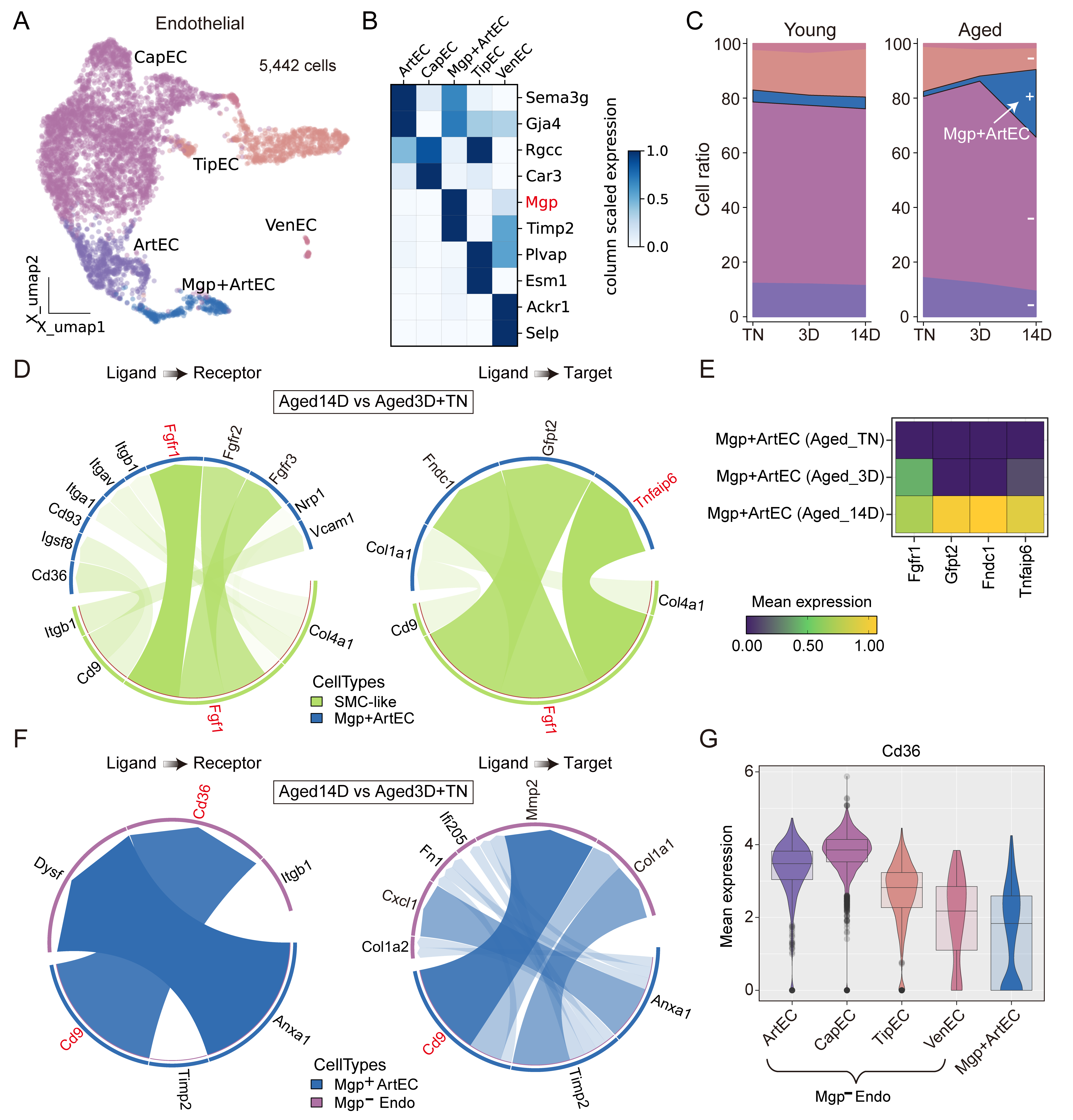


**Supplementary Figure S7:** **Age-related cross-talk between SMC-like and ECs following cold stimulation.** (A) UMAP plot showing the clustering distribution of 5,442 ECs from sWAT in young and aged mice, including capillary EC (CapEC), venous EC (VenEC), tip EC (TipEC), arterial EC (ArtEC), and Mgp+ ArtEC. (B) Heatmap displaying the expression levels of cell type-specific marker genes in endothelial subpopulations, with darker colors indicating higher expression. (C) Stacked bar plot illustrating the proportions of endothelial subpopulations under different conditions (TN, cold stimulation for 3 days, and 14 days). (D) Chord diagram showing ligand-receptor (target) pairs that were specifically upregulated in the interaction between SMC-like cells and Mgp+ ArtEC after 14 days of cold stimulation in aged mice. (E) Heatmap depicting the differential expression of receptor (Fgfr1) and target molecules (Gfpt2, Fndc1, and Tnfaip6) in Mgp+ ArtEC cells following cold exposure in aged mice. (F) Chord diagram illustrating ligand-receptor (target) pairs that were specifically upregulated in the interaction between Mgp+ ArtEC cells and other Mgp− endothelial subpopulations after 14 days of cold stimulation in aged mice. (G) Violin plot showing the differential expression of Cd36 across various ECs subpopulations.
